# Variants in the *FTO* and *CDKAL1* loci have recessive effects on risk of obesity and type 2 diabetes, respectively

**DOI:** 10.1007/s00125-016-3908-5

**Published:** 2016-03-10

**Authors:** Andrew R. Wood, Jessica Tyrrell, Robin Beaumont, Samuel E. Jones, Marcus A. Tuke, Katherine S. Ruth, The GIANT consortium, Hanieh Yaghootkar, Rachel M. Freathy, Anna Murray, Timothy M. Frayling, Michael N. Weedon

**Affiliations:** Genetics of Complex Traits, Institute of Biomedical and Clinical Science, University of Exeter Medical School, Royal Devon and Exeter Hospital, Barrack Road, Exeter, EX2 5DW UK

**Keywords:** Association analysis, BMI, *CDKAL1*, *FTO*, Genetics, Non-additive effects, Type 2 diabetes, UK Biobank

## Abstract

**Aims/hypothesis:**

Genome-wide association (GWA) studies have identified hundreds of common genetic variants associated with obesity and type 2 diabetes. These studies have usually focused on additive association tests. Identifying deviations from additivity may provide new biological insights and explain some of the missing heritability for these diseases.

**Methods:**

We performed a GWA study using a dominance deviation model for BMI, obesity (29,925 cases) and type 2 diabetes (4,040 cases) in 120,286 individuals of British ancestry from the UK Biobank study. We also investigated whether single nucleotide polymorphisms previously shown to be associated with these traits showed any enrichment for departures from additivity.

**Results:**

Known obesity-associated variants in *FTO* showed strong evidence of deviation from additivity (*p*_DOMDEV_ = 3 × 10^−5^) through a recessive effect of the allele associated with higher BMI. The average BMI of individuals carrying zero, one or two BMI-raising alleles was 27.27 (95% CI 27.22, 27.31) kg/m^2^, 27.54 (95% CI 27.50, 27.58) kg/m^2^ and 28.07 (95% CI 28.00, 28.14) kg/m^2^, respectively. A similar effect was observed in 105,643 individuals from the GIANT Consortium (*p*_DOMDEV_ = 0.003; meta-analysis *p*_DOMDEV_ = 1 × 10^−7^). For type 2 diabetes, we detected a recessive effect (*p*_DOMDEV_ = 5 × 10^−4^) at *CDKAL1*. Relative to homozygous non-risk allele carriers, homozygous risk allele carriers had an OR of 1.48 (95% CI 1.32, 1.65), while the heterozygous group had an OR of 1.06 (95% CI 0.99, 1.14), a result consistent with that of a previous study. We did not identify any novel associations at genome-wide significance.

**Conclusions/interpretation:**

Although we found no evidence of widespread non-additive genetic effects contributing to obesity and type 2 diabetes risk, we did find robust examples of recessive effects at the *FTO* and *CDKAL1* loci.

**Access to research materials:**

Summary statistics are available at www.t2diabetesgenes.org and by request (a.r.wood@exeter.ac.uk). All underlying data are available on application from the UK Biobank.

**Electronic supplementary material:**

The online version of this article (doi:10.1007/s00125-016-3908-5) contains peer-reviewed but unedited supplementary material, which is available to authorised users.

## Introduction

Genome-wide association (GWA) studies have identified hundreds of variants associated with obesity and type 2 diabetes [1–9]. However, GWA studies of type 2 diabetes and obesity have usually focused on testing additive models. An additive model assumes that the disease risk of heterozygous individuals is exactly halfway between those of the two homozygous groups. Non-additive effects include dominant and recessive effects. These effects are common in monogenic disorders, but there are only a few examples in common diseases and traits [10]. For obesity and type 2 diabetes, the strongest evidence of a non-additive effect is at the *CDKAL1* locus, where a previous study demonstrated a recessive effect [11]. The GIANT Consortium previously tested 32 BMI-associated variants for deviations from the additive model but, overall, found no evidence of deviation from additivity in 105,643 individuals [5].

There are at least three reasons why it is important to test for non-additive associations between common genetic variants and type 2 diabetes and obesity. First, a genome-wide approach that tests alternative models could identify new variants and candidate genes because the correct model may have more statistical power. Second, the correct model of inheritance could explain more of the variation in the trait, and hence account for some of the ‘missing heritability’ [12]. Third, the presence of recessive or dominant effects may inform follow-up physiological studies in vivo and in humans: for example, by prioritising recruit-by-genotype efforts on heterozygous as well as homozygous individuals.

The UK Biobank provides an excellent opportunity to test for deviation from additivity in a single large cohort, as genome-wide genetic data and detailed phenotypic data are available in the initial release of data from over 120,000 British individuals [13]. In this study we used the UK Biobank to perform GWA tests for deviations from the additive model for BMI, obesity and type 2 diabetes. We also investigated whether evidence of deviation was present for previously published single nucleotide polymorphisms (SNPs) associated with these traits.

## Methods

### Samples

We used the data of 120,286 individuals of British descent from the first UK Biobank genetic data release. Basic characteristics are given in electronic supplementary material (ESM) Table 1. British descent was defined as individuals who both self-identified as white British and were confirmed as ancestrally white using principal component analyses. Related individuals (third degree or higher) were estimated by the central UK Biobank team and removed to provide the maximal unrelated set of individuals. Details of principal component analyses and kinship analyses can be found in the official UK Biobank genotyping document at http://biobank.ctsu.ox.ac.uk/crystal/docs/genotyping_qc.pdf (accessed 1 December 2015).

### Genotypes

We used imputed genotypes available from the UK Biobank for association analyses. Briefly, phasing of individuals was carried out by UK Biobank using SHAPEIT version 2; imputation was performed using IMPUTE version 2 and a combined 1000 Genomes/UK10K reference panel. Full details can be found in the official UK Biobank imputation document at http://biobank.ctsu.ox.ac.uk/crystal/docs/impute_ukb_v1.pdf (accessed 1 December 2015). Using the data of 120,286 individuals for analysis, variants were excluded if imputation quality was <0.9, Hardy–Weinberg equilibrium was *p* < 1 × 10^−6^, or minor allele frequency (MAF) was <0.5%. This quality control process resulted in 9,288,881 variants for association analysis.

### Selection of known SNPs

#### BMI and obesity

We selected common genetic variants that were associated with BMI in the most recent meta-analysis from the GIANT Consortium [2, 5]. We limited the BMI SNPs to one per locus (defined as a 500 kb window) and those that were associated with BMI in the analysis of all European ancestry individuals. In total, 72 SNPs previously associated with BMI were analysed (ESM Table 2). This SNP list was used for both BMI and obesity analyses.

#### Type 2 diabetes

We selected common genetic variants previously associated with type 2 diabetes in the Diabetes Genetics Replication and Meta-Analysis (DIAGRAM) Consortium [3]. Details of the 66 type 2 diabetes SNPs are provided in ESM Table 3.

### Within-British principal component analysis

The UK Biobank study identified 120,286 individuals who were both self-identified as white British and confirmed as ancestrally white using genetics and principal component analyses. The 120,286 individuals excluded third-degree or closer relatives. We performed an additional round of principal component analysis on these 120,286 UK Biobank participants. We selected 95,535 independent SNPs (pairwise *r*^2^ < 0.1) directly genotyped with an MAF ≥2.5% and missingness <1.5% across all UK Biobank participants with genetic data available at the time of this study (*n* = 152,732), and with Hardy–Weinberg equilibrium *p* > 1 × 10^−6^ within the white British participants. Principal components were subsequently generated using fast principal component analysis of large-scale genome-wide data (flashpca) [14].

### Phenotypes

#### BMI

The UK Biobank provides two measures of BMI: one calculated from weight (kg)/height (m^2^) and one using electrical impedance. We excluded individuals with differences >4.56 SDs between impedance and normal BMI measures where both variables were available. If only one measure of BMI was available this was used. We corrected BMI by regressing age, sex, study centre and the first five within-British principal components, and taking residual values. We then inverse-normalised the residuals and used this phenotype for analysis. A total of 119,688 white British individuals with BMI and genetic data were available.

#### ‘Obese’ and ‘severely obese’ categorical variables

Individuals were classified as obese if their BMI was >30 kg/m^2^ (*N* = 29,925), and severely obese if their BMI was (>40 kg/m^2^) (*N* = 2,389). Controls for both were defined as those with a BMI <25 kg/m^2^.

#### Type 2 diabetes

Individuals were defined as having type 2 diabetes if they reported either type 2 diabetes or generic diabetes at the interview stage of the UK Biobank study. Individuals were excluded if they reported insulin use within the first year of diagnosis. Individuals reportedly diagnosed under the age of 35 years or with no known age of diagnosis were excluded, to limit the numbers of individuals with slow-progressing autoimmune diabetes or monogenic forms. Individuals diagnosed with diabetes within the last year of this study were also excluded as we were unable to determine whether they were using insulin within this time frame. A total of 4,040 cases and 113,735 controls within the white British subset of the UK Biobank with available genetic data were identified.

### Statistical analyses

#### Association testing

We adjusted for genotyping chip at run-time for the analyses of additive, dominance deviation, and recessive models for both BMI and type 2 diabetes. Association testing was performed through standard linear and logistic regression methods applied to BMI and type 2 diabetes, respectively. Logistic regression models included covariates at run-time. Type 2 diabetes was adjusted for age, sex and the first five within-British principal components.

#### Deviation from the additive model

If an allele operates through a purely additive model then statistical evidence of a dominant and/or recessive mechanism may still be detected given counts of the three genotype classes. Similarly, if an allele operates through a dominant or recessive mechanism an additive model may also detect it. To detect genuine differences between additive and non-additive effects, we performed a regression analysis against our traits of interest with a term representing the additive model for genotypes (coded 0, 1 and 2 for the homozygote, heterozygous and alternate homozygote groups, respectively) and a term representing the heterozygous group (coded 0, 1 and 0). This test (known as the ‘dominance deviation test’) determines whether the average trait value carried by the heterozygous groups lies halfway between the two homozygote groups as expected under an additive model.

#### Statistical threshold for GWA analysis

To determine a genome-wide significance threshold for genotypes available in the UK Biobank we first estimated the number of independent variants from those imputed with an imputation quality ≥0.4 and a minor allele count ≥5 within the 120,000 British UK Biobank individuals. We took a conservative pairwise *r*^2^ threshold of 0.9 and this resulted in 15,005,727 variants estimated as independent. A Bonferroni correction of this number resulted in a GWA *p* value threshold of 3 × 10^−9^.

#### Statistical thresholds for known SNP sets

When investigating previously published SNPs we applied a Bonferroni correction based on the number of SNPs (72 and 66 for BMI/obesity and type 2 diabetes, respectively). This resulted in a *p* value threshold of 7 × 10^−4^ and 8 × 10^−4^ for BMI/obesity status and type 2 diabetes, respectively.

#### Power calculations

Power calculations for BMI association were performed using QUANTO [15] based on sample size, variance explained and a significance level of 3 × 10^−9^. Calculations of equivalent power for type 2 diabetes were performed based on those of Yang et al [16].

### Ethics: UK Biobank

This study was conducted using the UK Biobank resource. Details of patient and public involvement in the UK Biobank are available online (www.ukbiobank.ac.uk/about-biobank-uk/ and www.ukbiobank.ac.uk/wp-content/uploads/2011/07/Summary-EGF-consultation.pdf?phpMyAdmin=trmKQlYdjjnQIgJ%2CfAzikMhEnx6). No patients were specifically involved in setting the research question or the outcome measures, nor were they involved in developing plans for recruitment, design or implementation of this study. No patients were asked to advise on interpretation or writing up of results. There are no specific plans to disseminate the results of the research to study participants, but the UK Biobank disseminates key findings from projects on its website.

## Results

### GWA study for deviation from additivity for BMI

We did not observe evidence of deviation from additivity at any SNP for BMI at our genome-wide significance level of *p* = 3 × 10^−9^. ESM Figure 1 presents the genome-wide QQ plot for the imputation-based dominance deviation test.

### Alleles at the *FTO* locus have a partially recessive effect on BMI and obesity status

Of the 72 known BMI variants, rs1421085, representing the signal at *FTO*, was the only one reaching *p*_DOMDEV_ < 7 × 10^−4^ (the Bonferroni threshold, given that we had tested 72 variants; Table 1 and ESM Table 4). This variant was recently identified as the best candidate causal variant at *FTO* [2, 17]. This variant is also in very strong linkage disequilibrium (*r*^2^ = 0.91, *D*′ > 0.99) with rs57292959, which was the variant with the third strongest evidence of deviation from additivity in the GWA analysis (MAF = 0.42; *p*_DOMDEV_ = 4 × 10^−7^).

**Table 1:** Summary statistics for the most strongly associated imputed SNP (rs57292959) and previously reported index SNP (rs1421085) at the *FTO* locus (*r* ^*2*^ = 0.91) with evidence of departure from the additive model

			Additive effects			Deviation from additivity		
SNP	Locus	Effect/other allele	*β*	SE	*p* value	*β*	SE	*p* value
rs1421085	*FTO*	C/T	0.076	0.004	2 × 10^−75^	−0.025	0.006	3 × 10^−5^
rs57292959	*FTO*	T/G	0.073	0.004	4 × 10^−68^	−0.030	0.006	4 × 10^−7^

Effect sizes are derived from BMI after inverse-normalisation of covariate-adjusted residuals

**Fig. 1:**
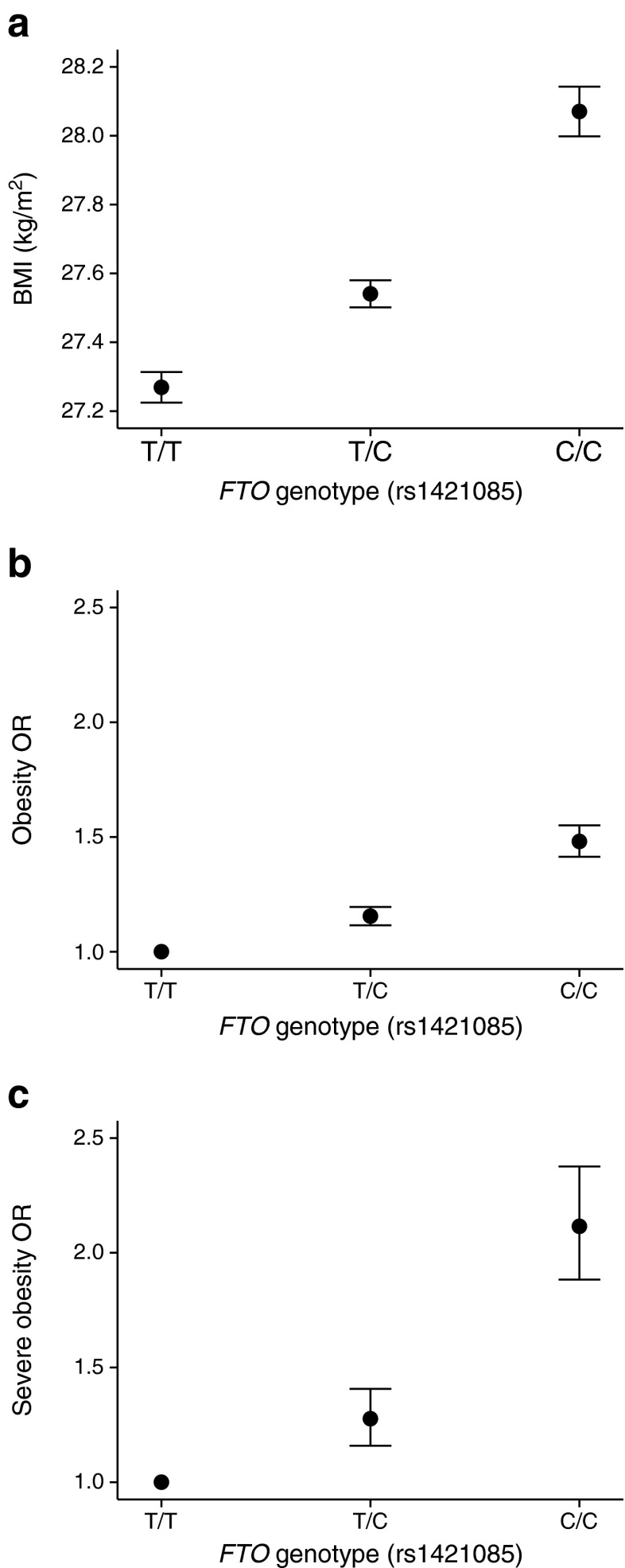
Average BMI and obesity ORs with 95% CIs for carriers of the BMI-raising allele at the *FTO* locus represented by rs1421085. (**a**) Average BMI within each of the three genotype classes. (**b**) Obesity risk for heterozygous and homozygous carriers of the BMI-increasing allele. (**c**) Severe obesity risk for the heterozygous and homozygous carriers of the BMI-increasing allele

Homozygous carriers of the previously reported BMI-raising allele (C at rs1421085) had an average BMI of 28.07 (95% CI 28.00, 28.14) kg/m^2^; heterozygotes had an average BMI of 27.54 (95% CI 27.50, 27.58) kg/m^2^; and homozygous carriers of the BMI-lowering allele had an average BMI of 27.27 (95% CI 27.22, 27.31) kg/m^2^ (Fig. 1a and Table 2). While heterozygous carriers were still on average more overweight than the common allele homozygote group, the difference (0.27 kg/m^2^) was approximately half of the difference observed between the heterozygote and minor allele homozygote groups (0.53 kg/m^2^). Accounting for this partially recessive effect only resulted in a small increase in variance in BMI (an additional 0.01%).

**Table 2:** BMI values by genotype group at the *FTO* locus (rs1421085)

rs1421085 genotype group	T/T	T/C	C/C
*n*	42,835	57,524	19,329
Mean (95% CI)	27.27 (27.22, 27.31)	27.54 (27.50, 27.58)	28.07 (28.00, 28.14)
SD	4.68	4.80	5.11

*N* = 119,688 white British individuals in the UK Biobank

The C allele is the BMI-raising allele

Units are in kg/m^2^

The *FTO* locus also showed a similar pattern of deviation from additivity in case–control analyses of obesity (*p*_DOMDEV_ = 0.001) and severe obesity (*p*_DOMDEV_ = 0.003) (Fig. 1b and c, and Table 3). The OR was stronger in homozygous carriers of the risk allele than expected under an additive model. The observed OR for obese heterozygous carriers of the BMI-raising allele was 1.15 (95% CI 1.12, 1.19). Under the additive model we would expect an OR ~1.32 for obese homozygous carriers, yet the observed OR was 1.48 (95% CI 1.41, 1.55). Similarly, the ORs observed in the severely obese heterozygote and homozygous carriers were 1.28 (95% CI 1.16, 1.41) and 2.12 (95% CI 1.88, 2.38), respectively, whereas under the additive model we would expect an OR ~1.64 for the homozygous carriers.

**Table 3:** ORs for ‘obese’ and ‘severely obese’ classifications by genotype group at the *FTO* locus (rs1421085)

Class	Additive		Dominance deviation from additivity		T/T vs T/C		T/C vs C/C		T/T vs C/C	
	OR (95% CI)	*p* value	OR (95% CI)	*p* value	OR (95% CI)	*p* value	OR (95% CI)	*p* value	OR (95% CI)	*p* value
Obese	1.20 (1.18, 1.23)	2 × 10^−60^	0.95 (0.92, 0.98)	0.001	1.15 (1.12, 1.19)	2 × 10^−16^	1.28 (1.23, 1.34)	3 × 10^−28^	1.48 (1.41, 1.55)	2 × 10^−62^
Severely obese	1.44 (1.36, 1.53)	2 × 10^−33^	0.88 (0.81, 0.96)	0.003	1.28 (1.16, 1.41)	7 × 10^−07^	1.66 (1.49, 1.85)	2 × 10^−20^	2.12 (1.88, 2.38)	2 × 10^−36^

The C allele is the risk-increasing allele

### The partially recessive effect at *FTO* is present in 105,643 individuals from the GIANT Consortium

The GIANT Consortium previously tested for deviations from additivity for 32 known BMI variants in 105,643 individuals [5]. There was no overall evidence of deviations from additivity at these known loci; however, *FTO* did have a similar partial recessive effect in this independent dataset, represented by rs1558902: another proxy of rs1421085 (*r*^2^ > 0.99, *D*′ = 1) (*p*_GIANT_DOMDEV_ = 0.003; *β* = −0.019; 95% CI −0.031, −0.008). The negative direction of effect for the heterozygous group in comparison with the two homozygous groups combined was consistent with that observed in the UK Biobank (Table 1) and indicative of a recessive effect for the BMI-increasing allele. Meta-analysing the studies strengthened the evidence of deviation from additivity (*N* = 225,143; *p*_META-ANALYSIS_ = 1 × 10^−7^).

### No evidence of non-additive effects at other known BMI variants

There was no evidence of deviation from additive effects for the remaining 71 BMI variants (ESM Table 4). Based on the 72 BMI variants, we also showed that using an inverse-normalised distribution of BMI produced very similar results to those using BMI on its naturally skewed scale (ESM Fig. 2).

### GWA study for deviation from additivity for type 2 diabetes

We did not identify any variants deviating from additivity for type 2 diabetes that reached genome-wide significance (ESM Fig. 3).

### Alleles at the *CDKAL1* locus have a recessive effect for type 2 diabetes

**Fig. 2:**
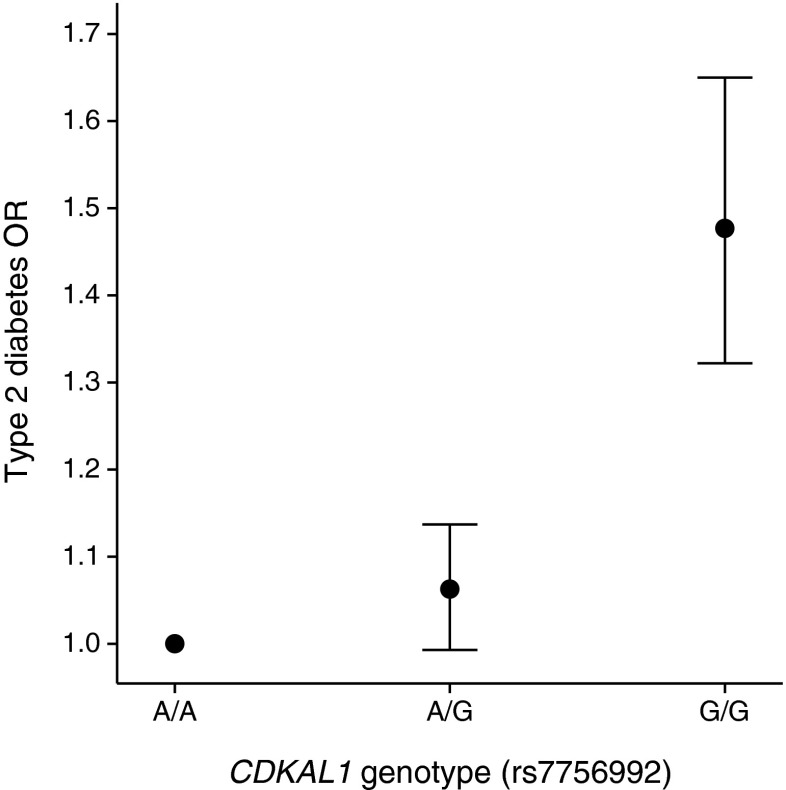
ORs and 95% CIs for heterozygous and homozygous carriers of the *CDKAL1* type 2 diabetes risk allele against the reference non-risk allele homozygous group

Of the 66 known type 2 diabetes variants we only found evidence of deviation from additivity for the SNP rs7756992 at the *CDKAL1* locus (MAF = 0.27; *p*_DOMDEV_ = 5 × 10^−4^) (Fig. 2, Table 4 and ESM Table 5). The genotype OR was stronger in homozygous carriers of the risk allele than expected under an additive model (Table 5). The observed OR within the heterozygous carriers of the risk-increasing allele was 1.06 (95% CI 0.99, 1.14; *p* = 0.08), which is smaller than the expected OR of ~1.22 for heterozygous carriers under an additive model. This finding is consistent with a previous study by deCODE genetics (Reykjavik, Iceland) that showed evidence of this SNP having a recessive pattern of association with type 2 diabetes risk [11]. We found no evidence of deviation from additivity for any of the remaining known type 2 diabetes variants.

**Table 4:** Summary statistics for rs7756992 at the *CDKAL1* locus showing evidence of deviation from additivity

			Additive effect			Dominance deviation		
SNP	Locus	Risk/other allele	OR	SE	*p* value	OR	SE	*p* value
rs7756992	*CDKAL1*	G/A	1.15	0.025	1 × 10^−8^	0.87	0.038	5 × 10^−4^

The effect allele is the allele observed to be the risk-raising allele under the additive test

**Table 5:** Type 2 diabetes ORs by genotype group at the *CDKAL1* locus (rs7756992)

Additive effect		A/A vs A/G		A/G vs G/G		A/A vs G/G	
OR (95% CI)	*p* value	OR (95% CI)	*p* value	OR (95% CI)	*p* value	OR (95% CI)	*p* value
1.15 (1.10, 1.21)	1 × 10^−08^	1.06 (0.99, 1.14)	0.077	1.39 (1.24, 1.56)	1 × 10^−8^	1.48 (1.32, 1.65)	6 × 10^−12^

*N* = 117,775 white British individuals in the UK Biobank

The G allele is the type 2 diabetes risk-increasing allele

## Discussion

Our analyses of 120,286 UK Biobank individuals suggest that most genetic variants associated with BMI and type 2 diabetes operate through a per-allele additive effect. Our findings suggest that dominant and recessive effects at common variants have a minimal role in explaining variation in BMI and risk of obesity and type 2 diabetes. Our results are consistent with a previous smaller study of 6,715 individuals that concluded that deviations from additivity contribute little to missing heritability for a wide range of traits [18]. There were exceptions for the *FTO*–BMI association and the *CDKAL1*–type 2 diabetes association.

The 16% of individuals carrying two copies of the BMI-raising allele at the *FTO* locus had more than twice the expected BMI difference compared with individuals carrying no BMI-raising alleles than would have been expected under a purely additive model. Assuming an average height in males of 1.78 m, it is equivalent to homozygous carriers of the BMI-increasing allele being 2.53 kg heavier than homozygous carriers of the opposite allele, whereas heterozygous carriers would be only 0.86 kg heavier. Previous studies have shown that the vast majority of this increased weight is fat mass [1]. The results are also consistent with a study of the *FTO* variant in polycystic ovary syndrome [19]. For type 2 diabetes, we found evidence of a recessive effect at the *CDKAL1* locus. The evidence that heterozygous carriers of the risk allele were at increased risk of type 2 diabetes was minimal and, combined with previous data from the deCODE study, suggests the true biological effect at this locus is recessive. Although accounting for non-additive effects at these loci only explained a small amount of additional variation in risk of obesity and type 2 diabetes, understanding why these associations demonstrate non-additivity may provide new insights into biological mechanisms at these loci.

A strength of our study is that we used a single large, relatively homogeneous dataset with full access to individual-level genotype and phenotype data. We had >80% power to detect dominance deviation from additivity, explaining 0.04% of the phenotypic variance at *p* = 3 × 10^−9^. This is equivalent to being able to detect a purely recessive effect of 0.4 kg/m^2^ for a BMI allele with a frequency of 0.25, for example. We had less power for the type 2 diabetes analysis (approximately ×6.5 less [16]). To have equivalent power to our BMI analysis we would require approximately 26,000 cases and 740,000 controls. Our analyses show how single large studies such as the UK Biobank will provide added value to existing meta-analyses approaches in GWA studies.

Our analyses have some limitations. We analysed imputed variants, and our statistical power to detect deviations from additivity might have been reduced if variants were not perfectly captured and/or we analysed imperfect markers for causal alleles. Non-biological explanations for the non-additive effects include ‘haplotype effects’ due to linkage disequilibrium with other causal alleles. In such situations, alleles of SNPs showing evidence of non-additivity are partially correlated with a much stronger causal SNP with an additive effect [20, 21]. This is unlikely to be the case at the *FTO* or *CDKAL1* loci. These loci have been studied extensively through re-sequencing and fine-mapping efforts and no substantially stronger individual variants have been identified; and for *FTO*, rs1421085 was recently proposed as the most likely causal variant [17]. The rs1421085 SNP disrupts a binding site for *ARID5B*, which increases the expression of *IRX3* and *IRX5* during adipocyte differentiation. This results in the production of more white adipocytes, a reduction in mitochondrial thermogenesis and an increase in lipid storage, although it is not clear why this would lead to non-additive effects on BMI.

The detection of non-additive genetic effects for BMI is potentially complicated by the skewed distribution of BMI. Effects that seem recessive could be artefacts of the skewed nature of the BMI distribution, as variation in BMI is wider towards the more overweight end of the distribution. To limit this effect we inverse-normalised BMI and performed additional sensitivity analyses (including ‘robust regression’—an alternative to ‘least squares regression’) that account for different variances of a trait, which may be the case for *FTO* [22] (data not shown), to limit the influence of the skewed distribution. We found, however, no evidence that BMI-increasing alleles were more likely to have recessive effects than BMI-lowering alleles (ESM Table 4 and ESM Fig. 4). Alternatively, artificially truncating the BMI distribution into a normal distribution could reduce the power to detect recessive effects of BMI-increasing alleles. However, we saw very little reduction in statistical confidence of known BMI associations when using the inverse-normalised scale compared with the natural BMI scale.

In conclusion, we have performed tests of deviation from additivity for BMI, obesity and type 2 diabetes. Overall, there was little evidence of dominant and recessive effects. However, we found replicable examples of non-additive effects at *FTO* on BMI and obesity, and at *CDKAL1* on type 2 diabetes risk. Recessive effects have implications for the mechanism of action of these loci but do not explain appreciably more of the ‘missing heritability’.

## Electronic supplementary material

Below is the link to the electronic supplementary material.

ESM Table 1 (PDF 65 kb)

ESM Table 2 (PDF 98 kb)

ESM Table 3 (PDF 96 kb)

ESM Table 4 (PDF 106 kb)

ESM Table 5 (PDF 100 kb)

ESM Figure 1 (PDF 259 kb)

ESM Figure 2 (PDF 257 kb)

ESM Figure 3 (PDF 276 kb)

ESM Figure 4 (PDF 79 kb)

ESM Giant Consortium (PDF 194 kb)

## Abbreviations

GWA: Genome-wide association
MAF: Minor allele frequency
SNP: Single nucleotide polymorphism
