# Supplementary material for: Variants in the FTO and CDKAL1 loci have recessive effects on risk of obesity and type 2 diabetes, respectively
Source: Diabetologia. 2016 Mar 10;59:1214–21. doi: 10.1007/s00125-016-3908-5 (PMC4869698; doi:10.1007/s00125-016-3908-5)
Supplement: Supplementary file 1 — (PDF 65 kb) [file 125_2016_3908_MOESM1_ESM.pdf]

ESM Table 1. Summary data of UK Biobank participants included in study. 'SD' = standard deviation, 'Min' = minimum characteristic value, 'Max' = maximum characteristic value.

		Summary Statistics within White British Subject Group								
**Characteristic**	**Subject Group**	**N**	**Mean**	**SD**	**Min**	**Max**	**Q1 Mean (SD)**	**Q2 Mean (SD)**	**Q3 Mean (SD)**	**Q4 Mean (SD)**
N	All	120,286	-	-	-	-	-	-	-	-
	Males	56,936	-	-	-	-	-	-	-	-
	Females	63,350	-	-	-	-	-	-	-	-
	T2D Cases	4,040	-	-	-	-	-	-	-	-
	T2D Cases Male	2,711	-	-	-	-	-	-	-	-
	T2D Cases Female	1,329	-	-	-	-	-	-	-	-
Age (years)	All	120,286	56.92	7.94	40	73	46.22 (3.31)	55.16 (2.00)	61.07 (1.36)	66.33 (1.76)
	Males	56,936	57.26	8.03	40	73	46.14 (3.33)	55.78 (2.29)	61.99 (1.40)	66.91 (1.49)
	Females	63,350	56.62	7.85	40	70	46.29 (3.30)	55.14 (2.00)	61.06 (1.36)	66.29 (1.75)
	T2D Cases	4,040	61.11	6.18	40	70	52.34 (4.06)	60.27 (1.38)	64.52 (1.11)	68.08 (0.90)
	T2D Cases Male	2,711	61.32	6.08	40	70	53.16 (4.16)	60.66 (1.12)	64.54 (1.11)	68.10 (0.91)
	T2D Cases Female	1,329	60.69	6.35	40	70	52.23 (4.16)	60.22 (1.38)	64.46 (1.11)	68.03 (0.88)
BMI	All	119,688	27.53	4.82	12.12	74.68	22.28 (1.51)	25.54 (0.75)	28.31 (0.90)	34.00 (3.91)
	Males	56,668	27.95	4.31	15.25	63.44	23.17 (1.51)	26.24 (0.68)	28.70 (0.79)	33.67 (3.46)
	Females	63,020	27.15	5.21	12.12	74.68	21.67 (1.41)	24.85 (0.76)	27.82 (1.02)	34.28 (4.27)
	T2D Cases	4,003	32.12	5.76	17.71	74.68	25.70 (1.90)	29.74 (0.89)	33.16 (1.16)	38.88 (4.36)
	T2D Cases Male	2,686	31.6	5.12	17.83	57.35	25.80 (1.82)	29.55 (0.80)	32.59 (1.02)	38.46 (3.74)
	T2D Cases Female	1,317	33.17	6.77	17.71	74.68	25.52 (2.04)	30.27 (1.14)	34.58 (1.41)	42.34 (4.77)
Height (cm)	All	120,085	168.78	9.2	75	205	157.73 (3.67)	165.46 (1.70)	171.94 (2.01)	181.88 (4.15)
	Males	56,833	175.69	6.75	75	205	167.44 (3.46)	174.02 (1.41)	178.41 (1.12)	184.52 (3.41)
	Females	63,252	162.57	6.19	126	190	154.85 (3.03)	160.54 (1.11)	164.86 (1.39)	171.16 (3.10)
	T2D Cases	4,029	170.17	8.98	143	196	158.27 (3.98)	167.86 (2.28)	174.38 (1.68)	181.83 (3.57)
	T2D Cases Male	2,704	174.52	6.64	153	196	166.40 (3.45)	172.53 (1.11)	176.84 (1.39)	183.38 (3.27)
	T2D Cases Female	1,325	161.29	6.12	143	183	153.74 (2.97)	159.55 (1.18)	163.29 (1.10)	169.56 (3.09)
