# Supplementary material for: Variants in the FTO and CDKAL1 loci have recessive effects on risk of obesity and type 2 diabetes, respectively
Source: Diabetologia. 2016 Mar 10;59:1214–21. doi: 10.1007/s00125-016-3908-5 (PMC4869698; doi:10.1007/s00125-016-3908-5)
Supplement: Supplementary file 2 — (PDF 98 kb) [file 125_2016_3908_MOESM2_ESM.pdf]

ESM Table 2. Summary data of 72 SNPs representing previously published BMI loci. 'Chr.' = chromosome, 'HG b37' = human genome build-37, 'HWE P' = Hardy-Weinberg *P*-value based on best-guess genotypes derived from imputation.

Published BMI Locus	SNP	Chr.	Position (HG b37)	Effect Allele	Other Allele	N	Effect Allele Frequency	Best-Guess HWE P	Imputation Quality
AGBL4	rs657452	1	49,589,847	A	G	116,549	0.391	0.016	0.987
ELAVL4	rs11583200	1	50,559,820	C	T	116,937	0.387	0.004	0.987
NEGR1	rs3101336	1	72,751,185	C	T	119,688	0.600	0.962	1.000
FPGT-TNNI3K	rs12566985	1	75,002,193	G	A	118,939	0.438	0.226	0.995
FUBP1	rs12401738	1	78,446,761	A	G	118,615	0.382	0.084	0.995
PTBP2	rs11165643	1	96,924,097	T	C	118,771	0.593	0.014	0.996
GNAT2	rs17024393	1	110,154,688	C	T	119,507	0.025	0.352	0.989
SEC16B	rs543874	1	177,889,480	G	A	119,688	0.209	0.507	1.000
NAV1	rs2820292	1	201,784,287	C	A	119,688	0.567	0.428	1.000
TMEM18	rs13021737	2	632,348	G	A	118,673	0.832	0.620	0.991
ADCY3	rs10182181	2	25,150,296	G	A	118,496	0.486	0.719	0.995
KCNK3	rs11126666	2	26,928,811	A	G	118,497	0.254	0.383	0.995
LINC01122	rs1016287	2	59,305,625	T	C	118,602	0.300	0.710	0.994
EHBP1	rs11688816	2	63,053,048	G	A	115,250	0.548	0.585	0.981
LRP1B	rs2121279	2	143,043,285	T	C	118,337	0.124	0.873	0.987
UBE2E3	rs1528435	2	181,550,962	T	C	119,321	0.622	0.676	0.997
ERBB4	rs7599312	2	213,413,231	G	A	114,229	0.737	0.573	0.973
RARB	rs6804842	3	25,106,437	G	A	116,777	0.576	1.0E-04	0.988
FHIT	rs2365389	3	61,236,462	C	T	117,861	0.593	0.345	0.993
GBE1	rs3849570	3	81,792,112	A	C	118,926	0.347	0.702	0.995
CADM2	rs13078960	3	85,807,590	G	T	118,362	0.200	0.677	0.992
RASA2	rs16851483	3	141,275,436	T	G	119,625	0.066	0.925	0.999
ETV5	rs1516725	3	185,824,004	C	T	118,998	0.864	0.350	0.995
GNPDA2	rs10938397	4	45,182,527	G	A	119,688	0.434	0.038	1.000

SLC39A8	rs13107325	4	103,188,709	T	C	119,688	0.074	0.069	1.000
HHIP	rs11727676	4	145,659,064	T	C	119,688	0.903	0.805	1.000
POC5	rs2112347	5	75,015,242	T	G	119,688	0.640	0.071	1.000
C6orf106	rs205262	6	34,563,164	G	A	119,212	0.268	0.763	0.997
TFAP2B	rs2207139	6	50,845,490	G	A	119,545	0.169	0.727	0.999
FOXO3	rs9400239	6	108,977,663	C	T	118,089	0.709	0.944	0.992
PARK2	rs13191362	6	163,033,350	A	G	118,665	0.876	0.884	0.990
HIP1	rs1167827	7	75,163,169	G	A	119,688	0.565	0.173	1.000
PMS2L11	rs2245368	7	76,608,143	C	T	119,688	0.167	0.320	1.000
HNF4G	rs17405819	8	76,806,584	T	C	119,360	0.702	0.934	0.998
RALYL	rs2033732	8	85,079,709	C	T	119,688	0.744	0.244	1.000
C9orf93	rs4740619	9	15,634,326	T	C	119,197	0.553	0.735	0.998
LINGO2	rs10968576	9	28,414,339	G	A	119,688	0.324	0.864	1.000
EPB41L4B	rs6477694	9	111,932,342	C	T	117,585	0.353	0.829	0.990
TLR4	rs1928295	9	120,378,483	T	C	119,680	0.571	0.403	1.000
LMX1B	rs10733682	9	129,460,914	A	G	109,718	0.471	0.325	0.958
GRID1	rs7899106	10	87,410,904	G	A	119,064	0.049	0.853	0.986
HIF1AN	rs17094222	10	102,395,440	C	T	114,710	0.206	0.935	0.969
NT5C2	rs11191560	10	104,869,038	C	T	119,685	0.076	0.069	1.000
TRIM66	rs4256980	11	8,673,939	G	C	118,229	0.657	0.723	0.993
BDNF	rs11030104	11	27,684,517	A	G	119,568	0.797	0.412	0.999
HSD17B12	rs2176598	11	43,864,278	T	C	119,688	0.246	0.436	1.000
MTCH2	rs3817334	11	47,650,993	T	C	119,688	0.408	0.417	1.000
CADM1	rs12286929	11	115,022,404	G	A	119,177	0.525	0.750	0.991
BCDIN3D	rs7138803	12	50,247,468	A	G	119,688	0.369	0.446	1.000
CLIP1	rs11057405	12	122,781,897	G	A	119,688	0.894	0.030	1.000
MTIF3	rs9581854	13	28,017,782	T	C	117,920	0.179	0.028	0.986
OLFM4	rs12429545	13	54,102,206	A	G	117,103	0.126	0.731	0.978
STXBP6	rs10132280	14	25,928,179	C	A	114,325	0.706	0.865	0.975

PRKD1	rs12885454	14	29,736,838	C	A	119,014	0.642	0.787	0.996
PRKD1	rs11847697	14	30,515,112	T	C	119,688	0.044	0.391	1.000
NRXN3	rs7141420	14	79,899,454	T	C	115,333	0.517	0.488	0.984
DMXL2	rs3736485	15	51,748,610	A	G	116,461	0.463	0.514	0.987
MAP2K5	rs16951275	15	68,077,168	T	C	119,450	0.775	0.325	0.998
NLRC3	rs758747	16	3,627,358	T	C	114,180	0.273	0.174	0.972
GPRC5B	rs12446632	16	19,935,389	G	A	119,655	0.858	0.896	1.000
SBK1	rs2650492	16	28,333,411	A	G	115,534	0.294	0.983	0.981
ATP2A1	rs3888190	16	28,889,486	A	C	119,259	0.403	0.585	0.998
FTO	rs1421085	16	53,800,954	C	T	119,688	0.402	0.938	1.000
RABEP1	rs1000940	17	5,283,252	G	A	119,056	0.300	0.005	0.996
RPTOR	rs12940622	17	78,615,571	G	A	119,227	0.559	0.756	0.998
C18orf8	rs1808579	18	21,104,888	C	T	119,194	0.517	0.269	0.998
GRP	rs7243357	18	56,883,319	T	G	118,054	0.827	0.297	0.990
MC4R	rs6567160	18	57,829,135	C	T	119,099	0.234	0.473	0.997
PGPEP1	rs17724992	19	18,454,825	A	G	117,156	0.735	0.125	0.983
KCTD15	rs29941	19	34,309,532	G	A	119,688	0.672	0.137	1.000
QPCTL	rs2287019	19	46,202,172	C	T	116,603	0.822	0.129	0.979
ZC3H4	rs3810291	19	47,569,003	A	G	119,688	0.677	0.198	1.000
