# Supplementary material for: Variants in the FTO and CDKAL1 loci have recessive effects on risk of obesity and type 2 diabetes, respectively
Source: Diabetologia. 2016 Mar 10;59:1214–21. doi: 10.1007/s00125-016-3908-5 (PMC4869698; doi:10.1007/s00125-016-3908-5)
Supplement: Supplementary file 3 — (PDF 96 kb) [file 125_2016_3908_MOESM3_ESM.pdf]

ESM Table 3. Summary data of 66 SNPs representing previously published T2D loci. 'Chr.' = chromosome, 'HG b37' = human genome build-37, 'HWE P' = Hardy-Weinberg *P*-value based on best-guess genotypes derived from imputation.

Published T2D Locus	SNP	Chr.	Position (HG b37)	Effect Allele	Other Allele	N	Effect Allele Frequency	Best-Guess HWE P	Imputation Quality
NOTCH2	rs10923931	1	120,517,959	T	G	117,775	0.108	0.811	1.000
PROX1	rs2075423	1	214,154,719	G	T	115,031	0.669	0.360	0.988
GCKR	rs780094	2	27,741,237	C	T	117,775	0.618	0.372	1.000
THADA	rs10203174	2	43,690,030	C	T	117,548	0.887	0.841	0.998
BCL11A	rs243088	2	60,568,745	T	A	115,701	0.452	0.333	0.991
RBMS1	rs7569522	2	161,346,447	A	G	113,920	0.453	0.967	0.984
GRB14	rs13389219	2	165,528,876	C	T	117,536	0.606	0.586	0.999
IRS1	rs2943640	2	227,093,585	C	A	117,775	0.652	0.949	1.000
PPARG	rs1801282	3	12,393,125	C	G	117,775	0.880	0.168	1.000
UBE2E2	rs1496653	3	23,454,790	A	G	117,606	0.795	0.535	0.999
PSMD6	rs12497268	3	64,090,363	G	C	114,798	0.826	0.943	0.979
ADAMTS9	rs6795735	3	64,705,365	C	T	117,775	0.595	0.366	1.000
ADCY5	rs11717195	3	123,082,398	T	C	117,125	0.751	0.249	0.996
IGF2BP2	rs4402960	3	185,511,687	T	G	117,775	0.315	0.579	1.000
ST64GAL1	rs17301514	3	186,613,409	A	G	116,082	0.104	0.195	0.977
MAEA	rs6819243	4	1,293,245	C	T	117,775	0.023	0.900	1.000
WFS1	rs4458523	4	6,289,986	G	T	117,318	0.597	0.368	0.998
ANKRD55	rs459193	5	55,806,751	G	A	117,775	0.747	0.410	1.000
ZBED3	rs6878122	5	76,427,311	G	A	117,266	0.319	0.399	0.997
CDKAL1	rs7756992	6	20,679,709	G	A	117,775	0.265	0.387	1.000
ZFAND3	rs4299828	6	38,177,667	A	G	116,536	0.803	0.109	0.991
KCNK16	rs3734621	6	39,304,211	C	A	117,663	0.026	0.689	0.993
DGKB	rs17168486	7	14,898,282	T	C	116,961	0.172	0.855	0.994
JAZF1	rs849135	7	28,196,413	G	A	117,640	0.498	0.712	0.999

GCK	rs10278336	7	44,245,363	A	G	117,505	0.575	0.671	0.999
GCC1	rs17867832	7	126,996,837	G	T	114,743	0.081	0.638	0.960
KLF14	rs13233731	7	130,437,689	G	A	116,963	0.505	0.216	0.997
ANK1	rs516946	8	41,519,248	C	T	117,775	0.764	0.533	1.000
TP53INP1	rs7845219	8	95,937,502	T	C	116,753	0.504	0.342	0.997
SLC30A8	rs3802177	8	118,185,025	G	A	117,775	0.689	0.020	1.000
GLIS3	rs10758593	9	4,292,083	A	G	117,775	0.397	0.094	1.000
PTPRD	rs16927668	9	8,369,533	T	C	117,110	0.202	0.040	0.996
CDKN2A/B	rs10811661	9	22,134,094	T	C	117,775	0.825	0.912	1.000
TLE4	rs17791513	9	81,905,590	A	G	117,162	0.939	0.064	0.991
TLE1	rs2796441	9	84,308,948	G	A	117,775	0.579	0.103	1.000
CDC123/CAMK1D	rs11257655	10	12,307,894	T	C	117,775	0.210	0.801	1.000
VPS26A	rs12242953	10	70,865,342	G	A	116,956	0.936	0.013	0.987
ZMIZ1	rs12571751	10	80,942,631	A	G	117,775	0.537	0.590	1.000
HHEX/IDE	rs1111875	10	94,462,882	C	T	117,775	0.590	0.793	1.000
TCF7L2	rs7903146	10	114,758,349	T	C	117,775	0.289	0.989	1.000
DUSP8	rs2334499	11	1,696,849	T	C	115,485	0.421	0.948	0.989
KCNQ1	rs163184	11	2,847,069	G	T	115,657	0.475	0.675	0.991
KCNJ11	rs5215	11	17,408,630	C	T	117,775	0.359	0.693	1.000
ARAP1 (CENTD2)	rs1552224	11	72,433,098	A	C	117,775	0.845	0.834	1.000
MTNR1B	rs10830963	11	92,708,710	G	C	117,775	0.275	0.549	1.000
CCND2	rs11063069	12	4,374,373	G	A	117,775	0.206	0.010	1.000
KLHDC5	rs10842994	12	27,965,150	C	T	117,775	0.803	0.061	1.000
HMGA2	rs2261181	12	66,212,318	T	C	116,532	0.095	0.853	0.986
TSPAN8/LGR5	rs7955901	12	71,433,293	C	T	115,638	0.444	0.349	0.992
HNF1A (TCF1)	rs12427353	12	121,426,901	G	C	115,452	0.811	0.887	0.984
SPRY2	rs1359790	13	80,717,156	G	A	117,775	0.711	0.392	1.000
C2CD4A	rs4502156	15	62,383,155	T	C	117,775	0.560	0.249	1.000
HMG20A	rs7177055	15	77,832,762	A	G	117,775	0.720	0.737	1.000

ZFAND6	rs11634397	15	80,432,222	G	A	117,775	0.661	0.643	1.000
AP3S2	rs2007084	15	90,345,335	A	G	113,145	0.080	0.827	0.942
PRC1	rs12899811	15	91,544,076	G	A	117,491	0.302	0.682	0.998
FTO	rs9936385	16	53,819,169	C	T	116,909	0.389	0.990	0.995
BCAR1	rs7202877	16	75,247,245	T	G	117,775	0.905	0.459	1.000
SRR	rs2447090	17	2,298,974	A	G	114,713	0.640	0.167	0.986
HNF1B (TCF2)	rs4430796	17	36,098,040	G	A	117,775	0.482	0.065	1.000
HNF1B (TCF2)	rs11651052	17	36,102,381	A	G	114,720	0.476	0.361	0.984
MC4R	rs12970134	18	57,884,750	A	G	117,775	0.268	0.375	1.000
CILP2	rs10401969	19	19,407,718	C	T	117,480	0.076	0.967	0.996
PEPD	rs8182584	19	33,909,710	T	G	117,775	0.374	0.379	1.000
GIPR	rs8108269	19	46,158,513	G	T	113,306	0.277	0.185	0.977
HNF4A	rs4812829	20	42,989,267	A	G	117,775	0.159	0.906	1.000
