# Supplementary material for: Variants in the FTO and CDKAL1 loci have recessive effects on risk of obesity and type 2 diabetes, respectively
Source: Diabetologia. 2016 Mar 10;59:1214–21. doi: 10.1007/s00125-016-3908-5 (PMC4869698; doi:10.1007/s00125-016-3908-5)
Supplement: Supplementary file 4 — (PDF 106 kb) [file 125_2016_3908_MOESM4_ESM.pdf]

ESM Table 4. Summary statistics for 72 published BMI SNPs tested for deviation from additivity for BMI. Results from the additive and recessive models are also presented. 'SE' = standard error.

			Additive Model			Dominant Model			Recessive Model			Dominance Deviation		
SNP	Locus	Effect/Other Allele	BETA	SE	P	BETA	SE	P	BETA	SE	P	BETA	SE	P
rs1421085	*FTO*	C/T	0.076	0.004	1.6E-75	0.083	0.006	1.6E-43	0.130	0.008	4.7E-62	-0.025	0.006	3.5E-05
rs758747	*NLRC3*	T/C	0.015	0.005	1.8E-03	0.011	0.006	0.066	0.045	0.011	6.8E-05	-0.019	0.007	0.010
rs1000940	*RABEP1*	G/A	0.011	0.004	0.013	0.019	0.006	9.6E-04	-0.002	0.01	0.863	0.017	0.007	0.012
rs11126666	*KCNK3*	A/G	0.002	0.005	0.679	-0.003	0.006	0.584	0.025	0.012	0.032	-0.019	0.008	0.015
rs3101336	*NEGR1*	C/T	0.027	0.004	7.0E-11	0.051	0.008	1.4E-10	0.027	0.006	6.6E-06	0.014	0.006	0.023
rs3810291	*ZC3H4*	A/G	0.028	0.004	1.6E-10	0.028	0.009	3.0E-03	0.039	0.006	2.7E-11	-0.014	0.007	0.037
rs10182181	*ADCY3*	G/A	0.033	0.004	5.7E-16	0.037	0.007	2.2E-08	0.052	0.007	1.8E-14	-0.011	0.006	0.058
rs10733682	*LMX1B*	A/G	0.019	0.004	8.6E-06	0.032	0.007	2.2E-06	0.018	0.007	0.014	0.011	0.006	0.062
rs4740619	*C9orf93*	T/C	0.017	0.004	4.3E-05	0.016	0.007	0.025	0.027	0.006	1.7E-05	-0.009	0.006	0.112
rs17405819	*HNF4G*	T/C	0.014	0.004	1.2E-03	0.033	0.010	1.0E-03	0.013	0.006	0.020	0.010	0.007	0.139
rs1528435	*UBE2E3*	T/C	0.014	0.004	6.7E-04	0.013	0.008	0.113	0.022	0.006	2.6E-04	-0.009	0.006	0.164
rs12940622	*RPTOR*	G/A	0.017	0.004	4.0E-05	0.018	0.007	0.017	0.026	0.006	2.9E-05	-0.008	0.006	0.188
rs6804842	*RARB*	G/A	0.009	0.004	0.037	0.006	0.008	0.431	0.015	0.006	0.014	-0.008	0.006	0.189
rs3888190	*ATP2A1*	A/C	0.024	0.004	7.8E-09	0.026	0.006	1.3E-05	0.041	0.008	2.1E-07	-0.008	0.006	0.210
rs7599312	*ERBB4*	G/A	0.020	0.005	3.6E-05	0.020	0.012	0.085	0.025	0.006	1.8E-05	-0.009	0.008	0.213
rs2033732	*RALYL*	C/T	0.002	0.005	0.689	0.014	0.012	0.224	-0.001	0.006	0.916	0.009	0.008	0.213
rs12566985	*FPGT-TNNI3K*	G/A	0.011	0.004	6.1E-03	0.010	0.006	0.095	0.021	0.007	3.6E-03	-0.007	0.006	0.216
rs13078960	*CADM2*	G/T	0.024	0.005	3.6E-06	0.024	0.006	6.6E-05	0.053	0.015	3.1E-04	-0.010	0.009	0.254
rs13191362	*PARK2*	A/G	0.027	0.006	1.3E-05	0.071	0.023	2.4E-03	0.027	0.007	8.8E-05	0.015	0.013	0.267
rs16951275	*MAP2K5*	T/C	0.032	0.005	6.2E-11	0.041	0.013	1.6E-03	0.038	0.006	9.0E-11	-0.009	0.008	0.295
rs2121279	*LRP1B*	T/C	0.006	0.006	0.325	0.008	0.007	0.226	-0.01	0.024	0.668	0.013	0.013	0.312
rs543874	*SEC16B*	G/A	0.049	0.005	2.2E-22	0.057	0.006	2.3E-21	0.069	0.014	1.0E-06	0.009	0.009	0.316

rs7243357	*GRP*	T/G	0.012	0.005	0.027	0.035	0.017	0.041	0.011	0.006	0.073	0.010	0.010	0.317
rs10132280	*STXBP6*	C/A	0.020	0.005	8.0E-06	0.025	0.011	0.020	0.026	0.006	8.9E-06	-0.006	0.007	0.362
rs12429545	*OLFM4*	A/G	0.028	0.006	6.9E-06	0.028	0.007	4.0E-05	0.068	0.023	3.5E-03	-0.012	0.013	0.364
rs6477694	*EPB41L4B*	C/T	0.007	0.004	0.084	0.007	0.006	0.260	0.016	0.009	0.063	-0.006	0.006	0.378
rs4256980	*TRIM66*	G/C	0.020	0.004	2.3E-06	0.036	0.009	6.9E-05	0.022	0.006	1.4E-04	0.006	0.006	0.388
rs2650492	*SBK1*	A/G	0.018	0.005	7.2E-05	0.019	0.006	1.1E-03	0.034	0.01	9.7E-04	-0.006	0.007	0.400
rs11165643	*PTBP2*	T/C	0.016	0.004	1.0E-04	0.027	0.008	4.5E-04	0.018	0.006	3.4E-03	0.005	0.006	0.401
rs205262	*C6orf106*	G/A	0.028	0.005	1.6E-09	0.034	0.006	4.7E-09	0.037	0.011	8.9E-04	0.006	0.007	0.430
rs11727676	*HHIP*	T/C	0.003	0.007	0.676	0.026	0.030	0.382	0.002	0.007	0.816	0.013	0.017	0.441
rs2245368	*PMS2L11*	C/T	0.021	0.005	8.4E-05	0.025	0.006	7.0E-05	0.026	0.017	0.141	0.008	0.010	0.447
rs2820292	*NAV1*	C/A	0.019	0.004	3.6E-06	0.023	0.007	1.8E-03	0.027	0.006	1.4E-05	-0.004	0.006	0.450
rs9581854	*MTIF3*	T/C	0.014	0.005	8.1E-03	0.017	0.006	6.0E-03	0.014	0.016	0.390	0.007	0.010	0.456
rs11191560	*NT5C2*	C/T	0.027	0.008	4.6E-04	0.029	0.008	3.4E-04	0.025	0.037	0.499	0.015	0.020	0.460
rs11057405	*CLIP1*	G/A	0.030	0.007	4.2E-06	0.039	0.027	0.145	0.033	0.007	3.6E-06	-0.010	0.015	0.498
rs10938397	*GNPDA2*	G/A	0.030	0.004	5.9E-13	0.036	0.006	7.9E-09	0.044	0.007	1.8E-09	-0.004	0.006	0.507
rs7138803	*BCDIN3D*	A/G	0.034	0.004	1.7E-15	0.043	0.006	2.5E-13	0.045	0.008	7.1E-08	0.004	0.006	0.509
rs7141420	*NRXN3*	T/C	0.018	0.004	2.5E-05	0.021	0.007	2.5E-03	0.025	0.007	1.3E-04	-0.004	0.006	0.526
rs6567160	*MC4R*	C/T	0.054	0.005	1.0E-28	0.062	0.006	3.6E-26	0.081	0.013	2.3E-10	0.005	0.008	0.533
rs13021737	*TMEM18*	G/A	0.058	0.005	1.8E-26	0.109	0.017	4.2E-10	0.062	0.006	2.4E-23	0.006	0.010	0.539
rs11688816	*EHBP1*	G/A	0.013	0.004	1.4E-03	0.016	0.007	0.032	0.019	0.006	2.7E-03	-0.004	0.006	0.555
rs2365389	*FHIT*	C/T	0.029	0.004	3.8E-12	0.044	0.008	1.6E-08	0.035	0.006	1.2E-08	0.003	0.006	0.563
rs12286929	*CADM1*	G/A	0.010	0.004	0.011	0.012	0.007	0.089	0.016	0.006	0.016	-0.003	0.006	0.575
rs1016287	*LINC01122*	T/C	0.019	0.004	2.2E-05	0.021	0.006	2.9E-04	0.033	0.01	1.0E-03	-0.004	0.007	0.592
rs13107325	*SLC39A8*	T/C	0.040	0.008	3.8E-07	0.040	0.008	1.1E-06	0.094	0.04	0.020	-0.011	0.022	0.593
rs29941	*KCTD15*	G/A	0.017	0.004	6.4E-05	0.023	0.009	0.015	0.022	0.006	1.4E-04	-0.003	0.007	0.612
rs17094222	*HIF1AN*	C/T	0.015	0.005	4.8E-03	0.015	0.006	0.013	0.03	0.015	0.040	-0.005	0.009	0.615
rs2176598	*HSD17B12*	T/C	0.023	0.005	1.3E-06	0.025	0.006	1.7E-05	0.041	0.012	6.6E-04	-0.004	0.008	0.630

rs1167827	*HIP1*	G/A	0.020	0.004	1.7E-06	0.025	0.007	6.3E-04	0.027	0.006	1.6E-05	-0.003	0.006	0.632
rs2112347	*POC5*	T/G	0.026	0.004	6.1E-10	0.036	0.009	3.2E-05	0.033	0.006	1.3E-08	-0.003	0.006	0.632
rs12446632	*GPRC5B*	G/A	0.028	0.006	2.1E-06	0.041	0.020	0.047	0.031	0.007	2.9E-06	-0.005	0.012	0.658
rs16851483	*RASA2*	T/G	0.028	0.008	7.8E-04	0.028	0.009	1.3E-03	0.069	0.044	0.116	-0.010	0.024	0.673
rs3849570	*GBE1*	A/C	0.012	0.004	5.9E-03	0.013	0.006	0.024	0.02	0.009	0.024	-0.003	0.006	0.685
rs2207139	*TFAP2B*	G/A	0.039	0.005	1.7E-12	0.041	0.006	3.8E-11	0.072	0.017	3.9E-05	-0.004	0.010	0.701
rs3817334	*MTCH2*	T/C	0.031	0.004	1.7E-13	0.037	0.006	6.3E-10	0.045	0.008	5.2E-09	-0.002	0.006	0.729
rs12401738	*FUBP1*	A/G	0.012	0.004	4.7E-03	0.016	0.006	8.3E-03	0.015	0.008	0.060	0.002	0.006	0.732
rs17724992	*PGPEP1*	A/G	0.023	0.005	1.3E-06	0.033	0.011	3.8E-03	0.027	0.006	4.7E-06	-0.002	0.007	0.745
rs3736485	*DMXL2*	A/G	0.013	0.004	2.3E-03	0.015	0.006	0.017	0.019	0.007	9.3E-03	-0.002	0.006	0.768
rs9400239	*FOXO3*	C/T	0.016	0.005	3.3E-04	0.027	0.010	8.8E-03	0.018	0.006	1.6E-03	0.002	0.007	0.777
rs2287019	*QPCTL*	C/T	0.035	0.005	7.8E-11	0.056	0.017	7.6E-04	0.039	0.006	3.8E-10	-0.003	0.010	0.778
rs17024393	*GNAT2*	C/T	0.070	0.013	5.5E-08	0.071	0.013	8.1E-08	0.165	0.108	0.126	-0.015	0.055	0.793
rs11030104	*BDNF*	A/G	0.040	0.005	7.0E-15	0.063	0.015	1.8E-05	0.045	0.006	1.2E-13	-0.002	0.009	0.794
rs1808579	*C18orf8*	C/T	0.021	0.004	1.9E-07	0.028	0.007	4.5E-05	0.029	0.007	1.1E-05	-0.001	0.006	0.843
rs11583200	*ELAVL4*	C/T	0.019	0.004	4.3E-06	0.025	0.006	4.2E-05	0.027	0.008	8.8E-04	0.001	0.006	0.860
rs7899106	*GRID1*	G/A	0.023	0.009	0.014	0.024	0.010	0.015	0.035	0.058	0.550	0.005	0.031	0.870
rs11847697	*PRKD1*	T/C	0.013	0.010	0.186	0.013	0.010	0.198	0.035	0.068	0.609	-0.005	0.035	0.886
rs1516725	*ETV5*	C/T	0.032	0.006	5.5E-08	0.055	0.022	0.011	0.035	0.007	1.4E-07	-0.002	0.012	0.898
rs1928295	*TLR4*	T/C	0.010	0.004	0.016	0.014	0.007	0.055	0.012	0.006	0.045	0.001	0.006	0.931
rs10968576	*LINGO2*	G/A	0.024	0.004	5.4E-08	0.028	0.006	9.2E-07	0.036	0.009	1.7E-04	0.000	0.007	0.960
rs12885454	*PRKD1*	C/A	0.014	0.004	7.1E-04	0.021	0.009	0.015	0.018	0.006	2.6E-03	0.000	0.006	0.967
rs657452	*AGBL4*	A/G	0.014	0.004	8.4E-04	0.018	0.006	3.7E-03	0.02	0.008	0.012	0.000	0.006	0.997
