# Supplementary material for: Variants in the FTO and CDKAL1 loci have recessive effects on risk of obesity and type 2 diabetes, respectively
Source: Diabetologia. 2016 Mar 10;59:1214–21. doi: 10.1007/s00125-016-3908-5 (PMC4869698; doi:10.1007/s00125-016-3908-5)
Supplement: Supplementary file 5 — (PDF 100 kb) [file 125_2016_3908_MOESM5_ESM.pdf]

ESM Table 5. Summary statistics for 66 published Type-2 diabetes SNPs tested for deviation from additivity for Type-2 diabetes risk. Results from the additive and recessive models are also presented. 'SE' = standard error.

			Additive Model			Dominant Model			Recessive Model			Dominance Deviation		
SNP	Locus	Effect/Other Allele	Odds Ratio	SE	P	Odds Ratio	SE	P	Odds Ratio	SE	P	Odds Ratio	SE	P
rs7756992	*CDKAL1*	G/A	1.153	0.025	1.3E-08	1.127	0.032	2.3E-04	1.439	0.055	2.6E-11	0.874	0.038	4.6E-04
rs11257655	*CDC123/CAMK1D*	T/C	1.118	0.027	4.4E-05	1.162	0.033	5.3E-06	1.057	0.077	0.471	1.104	0.047	0.036
rs6878122	*ZBED3*	G/A	1.035	0.024	0.153	1.071	0.033	0.034	0.981	0.054	0.713	1.073	0.037	0.058
rs6795735	*ADAMTS9*	C/T	1.008	0.023	0.733	0.960	0.043	0.349	1.042	0.034	0.223	0.940	0.034	0.067
rs12242953	*VPS26A*	G/A	1.002	0.047	0.966	0.715	0.205	0.100	1.020	0.050	0.697	0.817	0.113	0.074
rs12571751	*ZMIZ1*	A/G	1.080	0.023	7.7E-04	1.067	0.040	0.105	1.140	0.035	1.6E-04	0.947	0.033	0.094
rs10842994	*KLHDC5*	C/T	1.076	0.029	0.012	1.286	0.093	6.8E-03	1.065	0.034	0.065	1.096	0.055	0.095
rs516946	*ANK1*	C/T	1.091	0.027	1.5E-03	1.052	0.072	0.482	1.125	0.033	3.9E-04	0.929	0.046	0.108
rs7569522	*RBMS1*	A/G	1.062	0.023	0.011	1.116	0.037	2.8E-03	1.044	0.040	0.287	1.054	0.033	0.109
rs2075423	*PROX1*	G/T	1.085	0.025	9.8E-04	1.201	0.056	9.8E-04	1.080	0.033	0.019	1.058	0.038	0.136
rs3734621	*KCNK16*	C/A	1.129	0.069	0.078	1.148	0.070	0.049	0.303	1.009	0.237	2.103	0.509	0.144
rs16927668	*PTPRD*	T/C	1.014	0.029	0.630	0.999	0.034	0.982	1.123	0.080	0.145	0.932	0.049	0.154
rs1359790	*SPRY2*	G/A	1.102	0.026	1.7E-04	1.093	0.061	0.142	1.139	0.032	6.2E-05	0.947	0.040	0.177
rs4812829	*HNF4A*	A/G	1.097	0.031	2.5E-03	1.122	0.035	9.9E-04	1.044	0.102	0.676	1.084	0.060	0.178
rs7955901	*TSPAN8/LGR5*	C/T	1.010	0.023	0.683	1.038	0.035	0.288	0.979	0.041	0.605	1.045	0.033	0.178
rs243088	*BCL11A*	T/A	1.060	0.023	0.011	1.051	0.036	0.168	1.118	0.039	4.5E-03	0.957	0.033	0.184
rs7903146	*TCF7L2*	T/C	1.400	0.024	7.3E-45	1.524	0.033	1.3E-37	1.592	0.050	9.5E-21	1.048	0.036	0.185
rs7202877	*BCAR1*	T/G	1.147	0.041	8.3E-04	1.042	0.177	0.815	1.168	0.044	4.0E-04	0.881	0.098	0.195
rs1801282	*PPARG*	C/G	1.116	0.036	2.4E-03	1.447	0.155	0.017	1.111	0.040	8.1E-03	1.113	0.085	0.211
rs3802177	*SLC30A8*	G/A	1.131	0.025	1.2E-06	1.276	0.060	5.6E-05	1.137	0.032	7.0E-05	1.049	0.040	0.229
rs17301514	*ST64GAL1*	A/G	1.039	0.037	0.305	1.052	0.040	0.205	0.906	0.165	0.549	1.108	0.090	0.258
rs7177055	*HMG20A*	A/G	1.092	0.026	7.0E-04	1.089	0.062	0.172	1.123	0.032	3.6E-04	0.955	0.041	0.262

rs10278336	*GCK*	A/G	1.097	0.023	7.0E-05	1.105	0.043	0.021	1.145	0.034	5.8E-05	0.964	0.034	0.269
rs10830963	*MTNR1B*	G/C	1.113	0.025	1.9E-05	1.150	0.032	1.6E-05	1.127	0.058	0.040	1.044	0.039	0.272
rs2261181	*HMGA2*	T/C	1.203	0.037	5.3E-07	1.202	0.040	4.8E-06	1.606	0.141	8.1E-04	0.917	0.080	0.277
rs4299828	*ZFAND3*	A/G	1.021	0.029	0.470	0.962	0.083	0.637	1.036	0.034	0.300	0.946	0.051	0.278
rs17168486	*DGKB*	T/C	1.122	0.029	9.1E-05	1.122	0.034	8.2E-04	1.309	0.086	1.6E-03	0.946	0.052	0.292
rs2334499	*DUSP8*	T/C	1.043	0.023	0.071	1.074	0.035	0.041	1.034	0.042	0.436	1.033	0.033	0.326
rs2943640	*IRS1*	C/A	1.057	0.024	0.021	1.051	0.050	0.318	1.084	0.033	0.013	0.967	0.036	0.355
rs10811661	*CDKN2A/B*	T/C	1.214	0.032	1.3E-09	1.527	0.113	1.8E-04	1.223	0.036	2.1E-08	1.062	0.065	0.355
rs163184	*KCNQ1*	G/T	1.114	0.023	2.7E-06	1.177	0.038	1.6E-05	1.134	0.038	9.0E-04	1.030	0.033	0.361
rs13389219	*GRB14*	C/T	1.094	0.024	1.5E-04	1.171	0.047	7.6E-04	1.102	0.033	3.5E-03	1.031	0.035	0.371
rs4458523	*WFS1*	G/T	1.073	0.023	2.7E-03	1.077	0.045	0.096	1.108	0.033	2.2E-03	0.970	0.034	0.372
rs2796441	*TLE1*	G/A	1.055	0.023	0.022	1.105	0.043	0.021	1.054	0.034	0.125	1.030	0.033	0.376
rs11717195	*ADCY5*	T/C	1.058	0.027	0.036	1.149	0.071	0.052	1.055	0.033	0.101	1.039	0.045	0.391
rs9936385	*FTO*	C/T	1.152	0.023	1.1E-09	1.213	0.034	2.2E-08	1.195	0.043	3.1E-05	1.029	0.033	0.397
rs12427353	*HNF1A (TCF1)*	G/C	1.011	0.030	0.713	1.082	0.091	0.389	1.003	0.034	0.926	1.044	0.054	0.425
rs1552224	*ARAP1 (CENTD2)*	A/C	1.163	0.033	5.0E-06	1.412	0.121	4.5E-03	1.168	0.037	2.6E-05	1.055	0.069	0.437
rs11634397	*ZFAND6*	G/A	1.005	0.024	0.848	1.035	0.051	0.504	0.995	0.033	0.865	1.028	0.036	0.449
rs10401969	*CILP2*	C/T	1.084	0.042	0.054	1.081	0.045	0.082	1.315	0.190	0.149	0.929	0.104	0.478
rs6819243	*MAEA*	C/T	1.060	0.074	0.427	1.056	0.075	0.472	1.648	0.598	0.404	0.817	0.308	0.511
rs1111875	*HHEX/IDE*	C/T	1.142	0.024	1.6E-08	1.232	0.046	5.4E-06	1.169	0.033	2.9E-06	1.021	0.034	0.543
rs12497268	*PSMD6*	G/C	1.073	0.031	0.024	1.072	0.099	0.483	1.087	0.036	0.020	0.965	0.059	0.546
rs1496653	*UBE2E2*	A/G	1.128	0.029	3.9E-05	1.172	0.086	0.064	1.150	0.034	4.1E-05	0.969	0.052	0.549
rs8108269	*GIPR*	G/T	1.095	0.026	4.1E-04	1.120	0.033	6.2E-04	1.124	0.059	0.047	1.022	0.040	0.588
rs7845219	*TP53INP1*	T/C	1.089	0.023	2.0E-04	1.135	0.039	1.1E-03	1.108	0.036	5.0E-03	1.017	0.033	0.608
rs2447090	*SRR*	A/G	1.026	0.024	0.296	1.056	0.050	0.273	1.023	0.033	0.490	1.018	0.036	0.614
rs459193	*ANKRD55*	G/A	1.141	0.027	1.1E-06	1.273	0.072	8.7E-04	1.155	0.033	1.1E-05	1.023	0.046	0.618
rs5215	*KCNJ11*	C/T	1.074	0.024	2.5E-03	1.084	0.033	0.015	1.126	0.046	0.011	0.985	0.034	0.653

rs8182584	*PEPD*	T/G	1.023	0.024	0.325	1.022	0.033	0.517	1.049	0.046	0.303	0.985	0.034	0.658
rs12970134	*MC4R*	A/G	1.111	0.025	3.2E-05	1.124	0.032	3.1E-04	1.201	0.059	1.8E-03	0.984	0.039	0.676
rs10923931	*NOTCH2*	T/G	1.090	0.036	0.016	1.091	0.039	0.026	1.225	0.138	0.141	0.970	0.078	0.691
rs4402960	*IGF2BP2*	T/G	1.155	0.024	2.1E-09	1.195	0.033	4.6E-08	1.225	0.050	5.3E-05	1.014	0.036	0.697
rs13233731	*KLF14*	G/A	1.086	0.023	3.4E-04	1.127	0.039	2.1E-03	1.106	0.036	5.5E-03	1.012	0.033	0.709
rs780094	*GCKR*	C/T	1.092	0.024	2.1E-04	1.128	0.048	0.011	1.119	0.033	6.5E-04	0.991	0.035	0.805
rs10203174	*THADA*	C/T	1.132	0.038	1.1E-03	1.296	0.162	0.110	1.137	0.041	1.7E-03	1.022	0.090	0.806
rs17791513	*TLE4*	A/G	1.054	0.049	0.279	1.039	0.265	0.885	1.058	0.051	0.268	0.966	0.142	0.806
rs4502156	*C2CD4A*	T/C	1.031	0.023	0.188	1.049	0.041	0.248	1.036	0.035	0.311	1.007	0.033	0.832
rs849135	*JAZF1*	G/A	1.113	0.023	3.0E-06	1.160	0.039	1.2E-04	1.148	0.036	1.6E-04	1.007	0.032	0.837
rs10758593	*GLIS3*	A/G	1.030	0.023	0.198	1.041	0.034	0.229	1.038	0.044	0.391	1.006	0.034	0.852
rs17867832	*GCC1*	G/T	1.002	0.042	0.962	1.003	0.045	0.941	0.978	0.206	0.915	1.015	0.112	0.893
rs4430796	*HNF1B (TCF2)*	G/A	1.090	0.023	1.6E-04	1.118	0.037	2.9E-03	1.126	0.037	1.5E-03	0.996	0.032	0.894
rs2007084	*AP3S2*	A/G	1.005	0.043	0.900	1.005	0.045	0.914	1.024	0.206	0.910	0.992	0.112	0.944
rs11063069	*CCND2*	G/A	1.061	0.028	0.035	1.067	0.033	0.051	1.107	0.078	0.192	0.997	0.048	0.944
rs12899811	*PRC1*	G/A	1.043	0.025	0.089	1.050	0.032	0.132	1.070	0.055	0.220	0.997	0.038	0.945
rs11651052	*HNF1B (TCF2)*	A/G	1.090	0.023	2.1E-04	1.119	0.038	2.9E-03	1.125	0.038	2.1E-03	0.998	0.033	0.949
