# Supplementary material for: Variants in the FTO and CDKAL1 loci have recessive effects on risk of obesity and type 2 diabetes, respectively
Source: Diabetologia. 2016 Mar 10;59:1214–21. doi: 10.1007/s00125-016-3908-5 (PMC4869698; doi:10.1007/s00125-016-3908-5)

ESM Figure 1. Quantile-Quantile plot showing the association statistics of SNPs available for genome-wide association testing of deviation from additivity for BMI. λ = genomic control inflation factor.

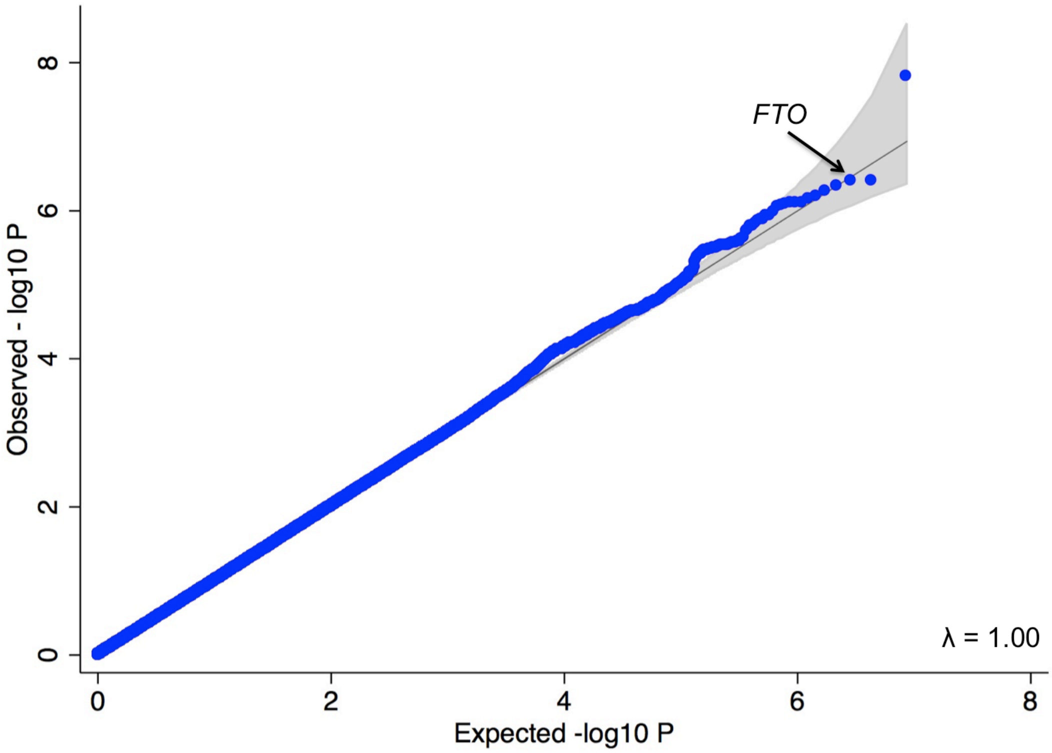

Supplement: Supplementary file 6 — (PDF 259 kb) [file 125_2016_3908_MOESM6_ESM.pdf]
