# Supplementary material for: Variants in the FTO and CDKAL1 loci have recessive effects on risk of obesity and type 2 diabetes, respectively
Source: Diabetologia. 2016 Mar 10;59:1214–21. doi: 10.1007/s00125-016-3908-5 (PMC4869698; doi:10.1007/s00125-016-3908-5)

ESM Figure 2. Association statistics for 72 known BMI variants when analysing BMI on an inverse-normalised scale (X axis) compared to its natural (right-hand skewed) scale (Y axis), under (**A**) additive, (**B**) recessive and (**C**) dominance deviation models.

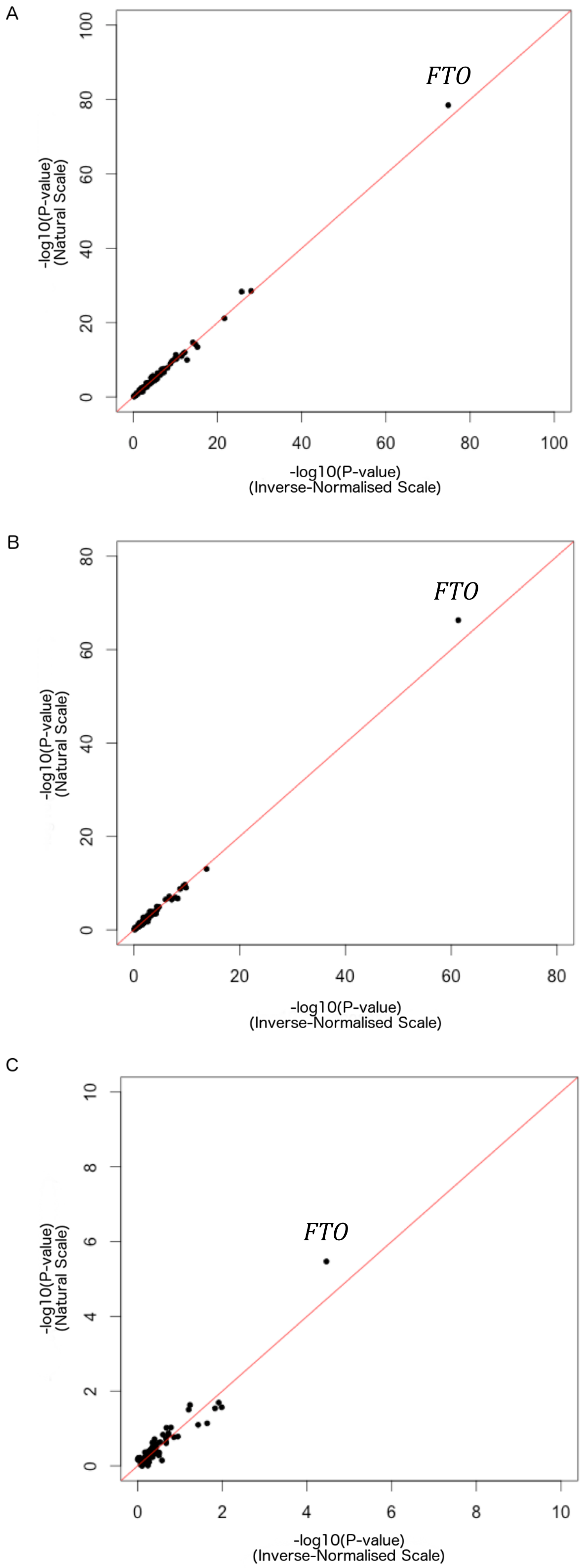

Supplement: Supplementary file 7 — (PDF 257 kb) [file 125_2016_3908_MOESM7_ESM.pdf]
