# Supplementary material for: Variants in the FTO and CDKAL1 loci have recessive effects on risk of obesity and type 2 diabetes, respectively
Source: Diabetologia. 2016 Mar 10;59:1214–21. doi: 10.1007/s00125-016-3908-5 (PMC4869698; doi:10.1007/s00125-016-3908-5)
Supplement: Supplementary file 9 — (PDF 79 kb) [file 125_2016_3908_MOESM9_ESM.pdf]

ESM Figure 4. Forrest plot showing deviation from additivity effects for 72 published BMI SNPs (aligned to the BMI increasing allele under an additive model). Overall *P*-value = 0.016 for testing whether BMI increasing alleles are more likely to have non-additive effects with the inclusion of *FTO*. Overall *P*-value without *FTO* = 0.072.

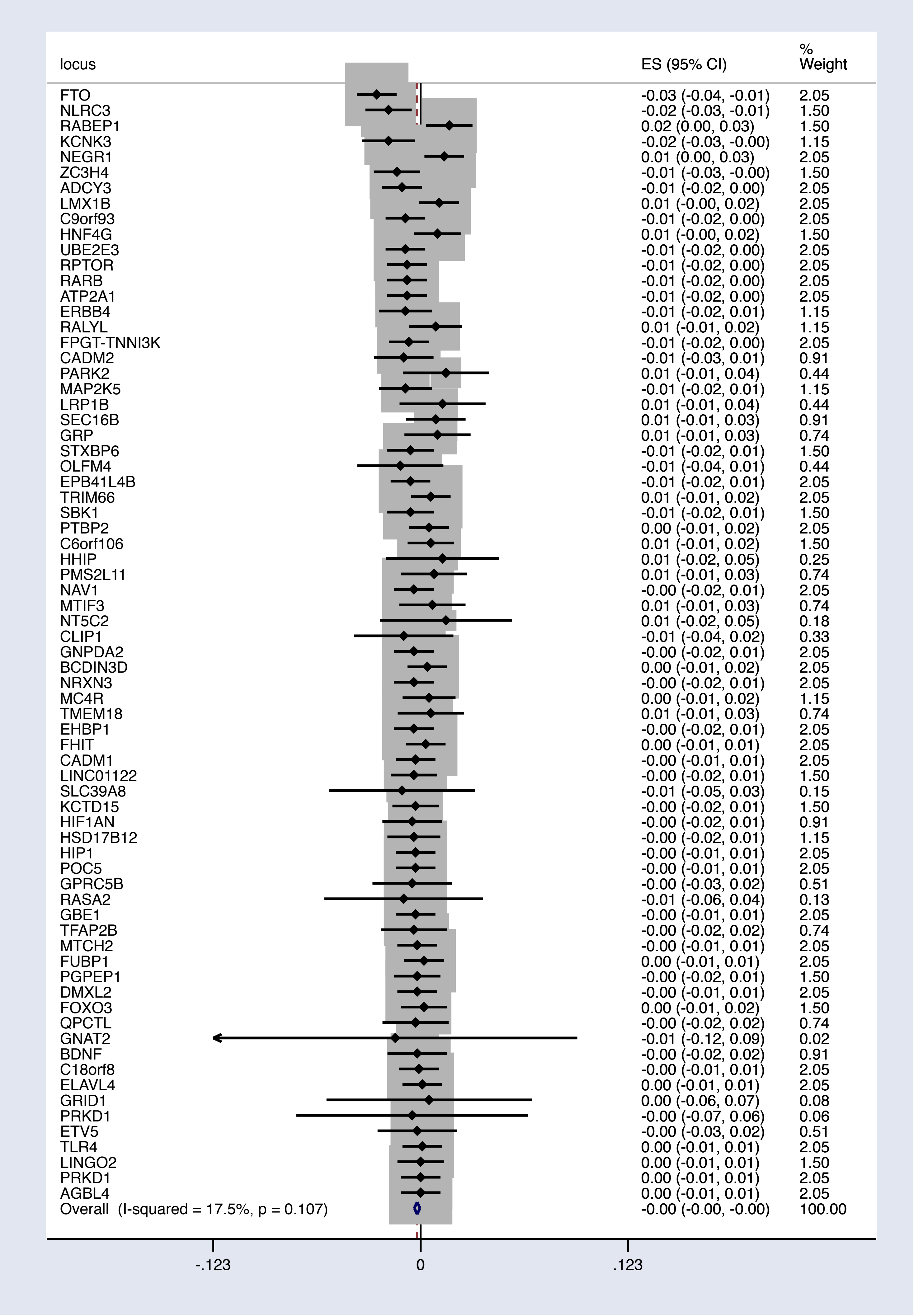

locus | ES (95% CI) | % Weight
FTO -0.03 (-0.04, -0.01) 2.05
NLRC3 -0.02 (-0.03, -0.01) 1.50
RABEP1 0.02 (0.00, 0.03) 1.50
KCNK3 -0.02 (-0.03, -0.00) 1.15
NEGR1 0.01 (0.00, 0.03) 2.05
ZC3H4 -0.01 (-0.03, -0.00) 1.50
ADCY3 -0.01 (-0.02, 0.00) 2.05
LMX1B 0.01 (-0.00, 0.02) 2.05
C9orf93 -0.01 (-0.02, 0.00) 2.05
HNF4G 0.01 (-0.00, 0.02) 1.50
UBE2E3 -0.01 (-0.02, 0.00) 2.05
RPTOR -0.01 (-0.02, 0.00) 2.05
RARB -0.01 (-0.02, 0.00) 2.05
ATP2A1 -0.01 (-0.02, 0.00) 2.05
ERBB4 -0.01 (-0.02, 0.01) 1.15
RALYL 0.01 (-0.01, 0.02) 1.15
FPGT-TNNI3K -0.01 (-0.02, 0.00) 2.05
CADM2 -0.01 (-0.03, 0.01) 0.91
PARK2 0.01 (-0.01, 0.04) 0.44
MAP2K5 -0.01 (-0.02, 0.01) 1.15
LRP1B 0.01 (-0.01, 0.04) 0.44
SEC16B 0.01 (-0.01, 0.03) 0.91
GRP 0.01 (-0.01, 0.03) 0.74
STXBP6 -0.01 (-0.02, 0.01) 1.50
OLFM4 -0.01 (-0.04, 0.01) 0.44
EPB41L4B -0.01 (-0.02, 0.01) 2.05
TRIM66 0.01 (-0.01, 0.02) 2.05
SBK1 -0.01 (-0.02, 0.01) 1.50
PTBP2 0.00 (-0.01, 0.02) 2.05
C6orf106 0.01 (-0.01, 0.02) 1.50
HHIP 0.01 (-0.02, 0.05) 0.25
PMS2L11 0.01 (-0.01, 0.03) 0.74
NAV1 -0.00 (-0.02, 0.01) 2.05
MTIF3 0.01 (-0.01, 0.03) 0.74
NT5C2 0.01 (-0.02, 0.05) 0.18
CLIP1 -0.01 (-0.04, 0.02) 0.33
GNPDA2 -0.00 (-0.02, 0.01) 2.05
BCDIN3D 0.00 (-0.01, 0.02) 2.05
NRXN3 -0.00 (-0.02, 0.01) 2.05
MC4R 0.00 (-0.01, 0.02) 1.15
TMEM18 0.01 (-0.01, 0.03) 0.74
EHBP1 -0.00 (-0.02, 0.01) 2.05
FHIT 0.00 (-0.01, 0.01) 2.05
CADM1 -0.00 (-0.01, 0.01) 2.05
LINC01122 -0.00 (-0.02, 0.01) 1.50
SLC39A8 -0.01 (-0.05, 0.03) 0.15
KCTD15 -0.00 (-0.02, 0.01) 1.50
HIF1AN -0.00 (-0.02, 0.01) 0.91
HSD17B12 -0.00 (-0.02, 0.01) 1.15
HIP1 -0.00 (-0.01, 0.01) 2.05
POC5 -0.00 (-0.01, 0.01) 2.05
GPRC5B -0.00 (-0.03, 0.02) 0.51
RASA2 -0.01 (-0.06, 0.04) 0.13
GBE1 -0.00 (-0.01, 0.01) 2.05
TFAP2B -0.00 (-0.02, 0.02) 0.74
MTCH2 -0.00 (-0.01, 0.01) 2.05
FUBP1 0.00 (-0.01, 0.01) 2.05
PGPEP1 -0.00 (-0.02, 0.01) 1.50
DMXL2 -0.00 (-0.01, 0.01) 2.05
FOXO3 0.00 (-0.01, 0.02) 1.50
QPCTL -0.00 (-0.02, 0.02) 0.74
GNAT2 -0.01 (-0.12, 0.09) 0.02
BDNF -0.00 (-0.02, 0.02) 0.91
C18orf8 -0.00 (-0.01, 0.01) 2.05
ELAVL4 0.00 (-0.01, 0.01) 2.05
GRID1 0.00 (-0.06, 0.07) 0.08
PRKD1 -0.00 (-0.07, 0.06) 0.06
ETV5 -0.00 (-0.03, 0.02) 0.51
TLR4 0.00 (-0.01, 0.01) 2.05
LINGO2 0.00 (-0.01, 0.01) 1.50
PRKD1 0.00 (-0.01, 0.01) 2.05
AGBL4 0.00 (-0.01, 0.01) 2.05
Overall (I-squared = 17.5%, p = 0.107) -0.00 (-0.00, -0.00) 100.00
-.123
0
.123
