# Supplementary material for: Variants in the FTO and CDKAL1 loci have recessive effects on risk of obesity and type 2 diabetes, respectively
Source: Diabetologia. 2016 Mar 10;59:1214–21. doi: 10.1007/s00125-016-3908-5 (PMC4869698; doi:10.1007/s00125-016-3908-5)
Supplement: Supplementary file 10 — (PDF 194 kb) [file 125_2016_3908_MOESM10_ESM.pdf]

GIANT Consortium 2010, Speliotes *et al.*

Elizabeth K Speliotes[1,2], Cristen J Willer[3], Sonja I Berndt[4] Keri LMonda[5], Gudmar Thorleifsson[6], Anne U Jackson[3], Hana Lango Allen[7], Cecilia M Lindgren[8,9], Jian'an Luan[10], Reedik Mägi[8], Joshua C Randall[8], Sailaja Vedantam[1,11], Thomas W Winkler[12], Lu Qi[13,14], Tsegaselassie Workalemahu[13], Iris M Heid[12,15], Valgerdur Steinthorsdottir[6], Heather M Stringham[3], Michael N Weedon[7], Eleanor Wheeler[16], Andrew R Wood[7], Teresa Ferreira[8], Robert J Weyant[3], Ayellet V Segrè[17–19], Karol Estrada[20–22], Liming Liang[23,24], James Nemesh[18], Ju-Hyun Park[4], Stefan Gustafsson[25], Tuomas OKilpeläinen[10], Jian Yang[26], Nabila Bouatia-Naji[27,28], Tõnu Esko[29–31], Mary F Feitosa[32], Zoltán Kutalik[33,34], Massimo Mangino[35], Soumya Raychaudhuri[18,36], Andre Scherag[37], Albert Vernon Smith[38,39,] Ryan Welch[3], Jing Hua Zhao[10], Katja KAben[40], Devin MAbsher[41], Najaf Amin[20], Anna LDixon[42], Eva Fisher[43], Nicole L Glazer[44,45], Michael E Goddard[46,47], Nancy L Heard-Costa[48], Volker Hoesel[49], Jouke-Jan Hottenga[50], Åsa Johansson[51,52], Toby Johnson[33,34,53,54], Shamika Ketkar[32], Claudia Lamina[15,55], Shengxu Li[10], Miriam F Moffatt[56], Richard H Myers[57], Narisu Narisu[58], John R B Perry[7], Marjolein J Peters[21,22], Michael Preuss[59], Samuli Ripatti[60,61], Fernando Rivadeneira[20–22], Camilla Sandholt[62], Laura J Scott[3], Nicholas J Timpson[63], Jonathan P Tyrer[64], Sophie van Wingerden[20], Richard M Watanabe[65,66], Charles C White[67], Fredrik Wiklund[25], Christina Barlassina[68], Daniel I Chasman[69,70], Matthew NCooper[71], John-Olov Jansson[72], Robert W Lawrence[71], Niina Pellikka[60,61], Inga Prokopenko[8,9], Jianxin Shi[4], Elisabeth Thiering[15], Helene Alavere[29], Maria T SAlibrandi[73], Peter Almgren[74,] Alice M Arnold[75,76], Thor Aspelund[38,39], Larry D Atwood[48], Beverley Balkau[77,78], Anthony J Balmforth[79], Amanda J Bennett[9], Yoav Ben-Shlomo[80], Richard N Bergman[66], Sven Bergmann[33,34], Heike Biebermann[81], Alexandra I F Blakemore[82], Tanja Boes[37], Lori L Bonnycastle[58], Stefan R Bornstein[83], Morris J Brown[84], Thomas A Buchanan[66,85], Fabio Busonero[86], Harry Campbell[87], Francesco P Cappuccio[88], Christine Cavalcanti-Proença[27,28], Yii-Der Ida Chen[89], Chih-Mei Chen[15], Peter S Chines[58], Robert Clarke[90], Lachlan Coin[91], John Connell[92], Ian N M Day[63], Martin den Heijer[93,94], Jubao Duan[95], Shah Ebrahim[96,97], Paul Elliott[91,98], Roberto Elosua[99], Gudny Eiriksdottir[38], Michael R Erdos[58], Johan GEriksson[100–104], Maurizio F Facheris[105,106], Stephan B Felix[107], Pamela Fischer-Posovszky[108], Aaron R Folsom[109], Nele Friedrich[110], Nelson B Freimer[111], Mao Fu[112], Stefan Gaget[27,28], Pablo V Gejman[95], Eco J C Geus[50], Christian Gieger[15], Anette P Gjesing[62], Anuj Goel[8,113], Philippe Goyette[114], Harald Grallert[15], Jürgen Gräßler[115], Danielle M Greenawalt[116], Christopher J Groves[9], Vilmundur Gudnason[38,39], Candace Guiducci[1], Anna-Liisa Hartikainen[117], Neelam Hassanali9, Alistair SHall[79], Aki SHavulinna[118], Caroline Hayward[119], Andrew C Heath[120], Christian Hengstenberg[121,122], Andrew A Hicks[105], Anke Hinney[123], Albert Hofman[20,22], Georg Homuth[124], Jennie Hui[71,125,126], Wilmar Igl[51], Carlos Iribarren[127,128], Bo Isomaa[103,129], Kevin B Jacobs[130], Ivonne Jarick[131], Elizabeth Jewell[3], Ulrich John[132], Torben Jørgensen[133,134], Pekka Jousilahti[118], Antti Jula[135], Marika Kaakinen[136,137], Eero Kajantie[101,138], Lee M Kaplan[2,70,139], Sekar Kathiresan[17,18,140–142], Johannes Kettunen[60,61], Leena Kinnunen[143], Joshua W Knowles[144], Ivana Kolcic[145], Inke R König[59], Seppo Koskinen[118], Peter Kovacs[146], Johanna Kuusisto[147], Peter Kraft[23,24], Kirsti Kvaløy[148], Jaana Laitinen[149], Olivier Lantieri[150], Chiara Lanzani[73], Lenore J Launer[151], Cecile Lecoeur[27,28], Terho Lehtimäki[152], Guillaume Lettre[114,153], Jianjun Liu[154], Marja-Liisa Lokki[155], Mattias Lorentzon[156], Robert N Luben[157], Barbara Ludwig[83], MAGIC[158], Paolo Manunta[73], Diana Marek[33,34], Michel Marre[159,160], Nicholas G Martin[161], Wendy L McArdle[162], Anne McCarthy[163], Barbara McKnight[75], Thomas Meitinger[164,165], Olle Melander[166,] David Meyre[27,28], Kristian Midthjell[148], Grant W Montgomery[167], Mario A Morken[58], Andrew P Morris[8], Rosanda Mulic[168], Julius S Ngwa[67], Mari Nelis[29–31], Matt J Neville[9], Dale R Nyholt[169], Christopher J O'Donnell[141,170], Stephen O'Rahilly[171], Ken K Ong[10], Ben Oostra[172], Guillaume Paré[173], Alex N Parker[174], Markus Perola[60,61], Irene Pichler[105], Kirsi H Pietiläinen[175,176], Carl GP Platou[148,177], Ozren Polasek[145,178], Anneli Pouta[117,179], Suzanne Rafelt[180], Olli Raitakari[181,182], Nigel W Rayner[8,9], Martin Ridderstråle[166], Winfried Rief[183], Aimo Ruokonen[184], Neil R

Robertson[8,9], Peter Rzehak[15,185], Veikko Salomaa[118], Alan R Sanders[95], Manjinder S Sandhu[10,16,157], Serena Sanna[86,] Jouko Saramies[186], Markku J Savolainen[187], Susann Scherag[123], Sabine Schipf[110,188], Stefan Schreiber[189], Heribert Schunkert[190], Kaisa Silander[60,61], Juha Sinisalo[191], David S Siscovick[45,192], Jan H Smit[193], Nicole Soranzo[16,35], Ulla Sovio[91], Jonathan Stephens[194,195], Ida Surakka[60,61], Amy J Swift[58], Mari-Liis Tammesoo[29], Jean-Claude Tardif[114,153], Maris Teder-Laving[30,31], Tanya M Teslovich[3], John R Thompson[196,197], Brian Thomson[1], Anke Tönjes[198,199], Tiinamaija Tuomi[103,200,201], Joyce B J van Meurs[20–22], Gert-Jan van Ommen[202,203], Vincent Vatin[27,28], Jorma Viikari[204], Sophie Visvikis-Siest[205], Veronique Vitart[119], Carla I G Vogel[123], Benjamin F Voight[17–19], Lindsay LWaite[41], Henri Wallaschofski[110], GBragi Walters[6], Elisabeth Widen[60], Susanna Wiegand[81], Sarah H Wild[87], Gonneke Willemsen[50], Daniel R Witte[206], Jacqueline CWitteman[20,22], Jianfeng Xu[207], Qunyuan Zhang[32], Lina Zgaga[145], Andreas Ziegler[59], Paavo Zitting[208], John P Beilby[125,126,209], I Sadaf Farooqi[171], Johannes Hebebrand[123], Heikki V Huikuri[210], Alan L James[126,211], Mika Kähönen[212], Douglas F Levinson[213], Fabio Macciardi[68,214], Markku SNieminen[191], Claes Ohlsson[156], Lyle J Palmer[71,126], Paul MRidker[69,70], Michael Stumvoll[198,215], Jacques S Beckmann[33,216], Heiner Boeing[43], Eric Boerwinkle[217], Dorret I Boomsma[50], Mark J Caulfield[54], Stephen J Chanock[4], Francis S Collins[58], L Adrienne Cupples[67], George Davey Smith[63], Jeanette Erdmann[190], Philippe Froguel[27,28,82], Henrik Grönberg[25], Ulf Gyllensten[51], Per Hall[25], Torben Hansen[62,218], Tamara B Harris[151], Andrew T Hattersley[7], Richard B Hayes[219], Joachim Heinrich[15], Frank B Hu[13,14,23], Kristian Hveem[148], Thomas Illig[15], Marjo-Riitta Jarvelin[91,136,137,179], Jaakko Kaprio[60,175,220], Fredrik Karpe[9,221], Kay-Tee Khaw[157], Lambertus A Kiemeney[40,93,222], Heiko Krude[81], Markku Laakso[147], Debbie A Lawlor[63], Andres Metspalu[29–31], Patricia B Munroe[54], Willem H Ouwehand[16,194,195], Oluf Pedersen[62,223,224], Brenda WPenninx[193,225,226], Annette Peters[15], Peter P Pramstaller[105,106,227], Thomas Quertermous[144], Thomas Reinehr[228], Aila Rissanen[176], Igor Rudan[87,168], Nilesh J Samani[180,196], Peter EH Schwarz[229], Alan R Shuldiner[112,230], Timothy D Spector[35], Jaakko Tuomilehto[143,231,232], Manuela Uda[86], André Uitterlinden[20–22], Timo T Valle[143], Martin Wabitsch[108], Gérard Waeber[233], Nicholas J Wareham[10], Hugh Watkins[8,113], on behalf of Procardis Consortium, James F Wilson[87], Alan F Wright[119], MCarola Zillikens[21,22], Nilanjan Chatterjee[4], Steven A McCarroll[17–19], Shaun Purcell[17,234,235], Eric E Schadt[236,237], Peter M Visscher[26], Themistocles L Assimes[144], Ingrid B Borecki[32,238], Panos Deloukas[16], Caroline S Fox[239], Leif C Groop[74], Talin Haritunians[89], David J Hunter[13,14,23], Robert C Kaplan[240], Karen L Mohlke[241], Jeffrey R O'Connell[112], Leena Peltonen[16,60,61,234,242], David Schlessinger[243], David P Strachan[244], Cornelia Mvan Duijn[20,22], H-Erich Wichmann[15,185,245], Timothy M Frayling7, Unnur Thorsteinsdottir[6,246], Gonçalo R Abecasis[3], Inês Barroso[16,247], Michael Boehnke[3], Kari Stefansson[6,246], Kari E North[5,248], Mark I McCarthy[8,9,221], Joel N Hirschhorn[1,11,249], Erik Ingelsson[25] & Ruth J F Loos[10]

[1]Metabolism Initiative and Program in Medical and Population Genetics, Broad Institute, Cambridge, Massachusetts, USA. [2]Division of Gastroenterology, Massachusetts General Hospital, Boston, Massachusetts, USA. [3]Department of Biostatistics, Center for Statistical Genetics, University of Michigan, Ann Arbor, Michigan, USA. [4]Division of Cancer Epidemiology and Genetics, National Cancer Institute, National Institutes of Health, Department of Health and Human Services, Bethesda, Maryland, USA. [5]Department of Epidemiology, School of Public Health, University of North Carolina at Chapel Hill, Chapel Hill, North Carolina, USA. [6]deCODE Genetics, Reykjavik, Iceland. [7]Genetics of Complex Traits, Peninsula College of Medicine and Dentistry, University of Exeter, Exeter, UK. [8]Wellcome Trust Centre for Human Genetics, University of Oxford, Oxford, UK. [9]Oxford Centre for Diabetes, Endocrinology and Metabolism, University of Oxford, Oxford, UK. [10]Medical Research Council (MRC) Epidemiology Unit, Institute of Metabolic Science, Addenbrooke's Hospital, Cambridge, UK. [11]Divisions of Genetics and Endocrinology and Program in Genomics, Children's Hospital, Boston, Massachusetts, USA. [12]Regensburg University Medical Center, Department of Epidemiology and Preventive Medicine, Regensburg, Germany. [13]Department of Nutrition, Harvard School of Public Health, Boston,

Massachusetts, USA. [14]Channing Laboratory, Department of Medicine, Brigham and Women's Hospital and Harvard Medical School, Boston, Massachusetts, USA. [15]Institute of Epidemiology, Helmholtz Zentrum München-German Research Center for Environmental Health, Neuherberg, Germany. [16]Wellcome Trust Sanger Institute, Hinxton, Cambridge, UK. [17]Center for Human Genetic Research, Massachusetts General Hospital, Boston, Massachusetts, USA. [18]Program in Medical and Population Genetics, Broad Institute of Harvard and Massachusetts Institute of Technology, Cambridge, Massachusetts, USA. [19]Department of Molecular Biology, Massachusetts General Hospital, Boston, Massachusetts, USA. [20]Department of Epidemiology, Erasmus Medical Center (MC), Rotterdam, The Netherlands. [21]Department of Internal Medicine, Erasmus MC, Rotterdam, The Netherlands. [22]Netherlands Genomics Initiative (NGI)-sponsored Netherlands Consortium for Healthy Aging (NCHA), Rotterdam, The Netherlands. [23]Department of Epidemiology, Harvard School of Public Health, Boston, Massachusetts, USA. [24]Department of Biostatistics, Harvard School of Public Health, Boston, Massachusetts, USA. [25]Department of Medical Epidemiology and Biostatistics, Karolinska Institutet, Stockholm, Sweden. [26]Queensland Statistical Genetics Laboratory, Queensland Institute of Medical Research, Queensland, Australia. [27]Centre National de la Recherche Scientifique (CNRS) UMR8199-IBL-Institut Pasteur de Lille, Lille, France. [28]University Lille Nord de France, Lille, France. [29]Estonian Genome Center, University of Tartu, Tartu, Estonia. [30]Estonian Biocenter, Tartu, Estonia. [31]Institute of Molecular and Cell Biology, University of Tartu, Tartu, Estonia. [32]Department of Genetics, Washington University School of Medicine, St. Louis, Missouri, USA. [33]Department of Medical Genetics, University of Lausanne, Lausanne, Switzerland. [34]Swiss Institute of Bioinformatics, Lausanne, Switzerland. [35]Department of Twin Research and Genetic Epidemiology, King's College London, London, UK. [36]Division of Rheumatology, Immunology and Allergy, Brigham and Women's Hospital, Harvard Medical School, Boston, Massachusetts, USA. [37]Institute for Medical Informatics, Biometry and Epidemiology, University of Duisburg-Essen, Essen, Germany. [38]Icelandic Heart Association, Kopavogur, Iceland. [39]University of Iceland, Reykjavik, Iceland. [40]Comprehensive Cancer Center East, Nijmegen, The Netherlands. [41]Hudson Alpha Institute for Biotechnology, Huntsville, Alabama, USA. [42]Department of Pharmacy and Pharmacology, University of Bath, Bath, UK. [43]Department of Epidemiology, German Institute of Human Nutrition Potsdam-Rehbruecke, Nuthetal, Germany. [44]Department of Medicine, University of Washington, Seattle, Washington, USA. [45]Cardiovascular Health Research Unit, University of Washington, Seattle, Washington, USA. [46]University of Melbourne, Parkville, Australia. [47]Department of Primary Industries, Melbourne, Victoria, Australia. [48]Department of Neurology, Boston University School of Medicine, Boston, Massachusetts, USA. [49]Technical University Munich, Chair of Biomathematics, Garching, Germany. [50]Department of Biological Psychology, Vrije Universiteit (VU) University Amsterdam, Amsterdam, The Netherlands. [51]Department of Genetics and Pathology, Rudbeck Laboratory, University of Uppsala, Uppsala, Sweden. [52]Department of Cancer Research and Molecular Medicine, Faculty of Medicine, Norwegian University of Science and Technology (NTNU), Trondheim, Norway. [53]Clinical Pharmacology, William Harvey Research Institute, Barts and The London School of Medicine and Dentistry, Queen Mary, University of London, London, UK. [54]Clinical Pharmacology and Barts and The London Genome Centre, William Harvey Research Institute, Barts and The London School of Medicine and Dentistry, Queen Mary University of London, Charterhouse Square, London, UK. [55]Division of Genetic Epidemiology, Department of Medical Genetics, Molecular and Clinical Pharmacology, Innsbruck Medical University, Innsbruck, Austria. [56]National Heart and Lung Institute, Imperial College London, London, UK. [57]Department of Neurology, Boston University School of Medicine, Boston, Massachusetts, USA. [58]National Human Genome Research Institute, National Institutes of Health, Bethesda, Maryland, USA. [59]Institut für Medizinische Biometrie und Statistik, Universität zu Lübeck, Universitätsklinikum Schleswig-Holstein, Campus Lübeck, Lübeck, Germany. [60]Institute for Molecular Medicine Finland (FIMM), University of Helsinki, Helsinki, Finland. [61]National Institute for Health and Welfare, Department of Chronic Disease Prevention, Unit of Public Health Genomics, Helsinki, Finland. [62]Hagedorn Research Institute, Gentofte, Denmark. [63]MRC Centre for Causal Analyses in Translational Epidemiology, Department of Social Medicine, Oakfield House, Bristol, UK. [64]Department of Oncology, University of Cambridge, Cambridge, UK. [65]Department of Preventive Medicine, Keck School of Medicine, University of Southern California, Los Angeles, California, USA. [66]Department of Physiology and Biophysics, Keck School of Medicine, University of Southern California, Los Angeles, California, USA. [67]Department of Biostatistics, Boston University School of Public Health, Boston, Massachusetts, USA. [68]University of Milan, Department of Medicine, Surgery and Dentistry,

Milano, Italy. [69]Division of Preventive Medicine, Brigham and Women's Hospital, Boston, Massachusetts, USA. [70]Harvard Medical School, Boston, Massachusetts, USA. [71]Centre for Genetic Epidemiology and Biostatistics, University of Western Australia, Crawley, Western Australia, Australia. [72]Department of Physiology, Institute of Neuroscience and Physiology, Sahlgrenska Academy, University of Gothenburg, Gothenburg, Sweden. [73]University Vita-Salute San Raffaele, Division of Nephrology and Dialysis, Milan, Italy. [74]Lund University Diabetes Centre, Department of Clinical Sciences, Lund University, Malmö, Sweden. [75]Department of Biostatistics, University of Washington, Seattle, Washington, USA. [76]Collaborative Health Studies Coordinating Center, Seattle, Washington, USA. [77]INSERM Centre de recherche en Epidémiologie et Santé des Populations (CESP) Centre for Research in Epidemiology and Public Health U1018, Villejuif, France. [78]University Paris Sud 11, Unité Mixte de Recherche en Santé (UMRS) 1018, Villejuif, France. [79]Multidisciplinary Cardiovascular Research Centre (MCRC), Leeds Institute of Genetics, Health and Therapeutics (LIGHT), University of Leeds, Leeds, UK. [80]Department of Social Medicine, University of Bristol, Bristol, UK. [81]Institute of Experimental Paediatric Endocrinology, Charité Universitätsmedizin Berlin, Berlin, Germany. [82]Department of Genomics of Common Disease, School of Public Health, Imperial College London, London, UK. [83]Department of Medicine III, University of Dresden, Dresden, Germany. [84]Clinical Pharmacology Unit, University of Cambridge, Addenbrooke's Hospital, Cambridge, UK. [85]Division of Endocrinology, Keck School of Medicine, University of Southern California, Los Angeles, California, USA. [86]Istituto di Neurogenetica e Neurofarmacologia del Consiglio Nazionale delle Ricerche (CNR), Monserrato, Cagliari, Italy. [87]Centre for Population Health Sciences, University of Edinburgh, Teviot Place, Edinburgh, Scotland, UK. [88]University of Warwick, Warwick Medical School, Coventry, UK. [89]Medical Genetics Institute, Cedars-Sinai Medical Center, Los Angeles, California, USA. [90]Clinical Trial Service Unit, Oxford, UK. [91]Department of Epidemiology and Biostatistics, School of Public Health, Faculty of Medicine, Imperial College London, London, UK. [92]University of Dundee, Ninewells Hospital and Medical School, Dundee, UK. [93]Department of Epidemiology, Biostatistics and HTA, Radboud University Nijmegen Medical Centre, Nijmegen, The Netherlands. [94]Department of Endocrinology, Radboud University Nijmegen Medical Centre, Nijmegen, The Netherlands. [95]Northshore University Healthsystem, Evanston, Illinois, USA. [96]The London School of Hygiene and Tropical Medicine, London, UK. [97]South Asia Network for Chronic Disease, New Dehli, India. [98]MRC-Health Protection Agency (HPA) Centre for Environment and Health, London, UK. [99]Cardiovascular Epidemiology and Genetics, Institut Municipal D'investigacio Medica and Centro de Investigación Biomédica en Red CIBER Epidemiología y Salud Pública, Barcelona, Spain. [100]Department of General Practice and Primary Health Care, University of Helsinki, Helsinki, Finland. [101]National Institute for Health and Welfare, Helsinki, Finland. [102]Helsinki University Central Hospital, Unit of General Practice, Helsinki, Finland. [103]Folkhalsan Research Centre, Helsinki, Finland. [104]Vasa Central Hospital, Vasa, Finland. [105]Institute of Genetic Medicine, European Academy Bozen-Bolzano (EURAC), Bolzano-Bozen, Italy, Affiliated Institute of the University of Lübeck, Lübeck, Germany. [106]Department of Neurology, General Central Hospital, Bolzano, Italy. [107]Department of Internal Medicine B, Ernst-Moritz-Arndt University, Greifswald, Germany. [108]Pediatric Endocrinology, Diabetes and Obesity Unit, Department of Pediatrics and Adolescent Medicine, Ulm, Germany. [109]Division of Epidemiology and Community Health, School of Public Health, University of Minnesota, Minneapolis, Minnesota, USA. [110]Institut für Klinische Chemie und Laboratoriumsmedizin, Universität Greifswald, Greifswald, Germany. [111]Center for Neurobehavioral Genetics, University of California, Los Angeles, California, USA. [112]Department of Medicine, University of Maryland School of Medicine, Baltimore, Maryland, USA. [113]Department of Cardiovascular Medicine, University of Oxford, John Radcliffe Hospital, Headington, Oxford, UK. [114]Montreal Heart Institute, Montreal, Quebec, Canada. [115]Department of Medicine III, Pathobiochemistry, University of Dresden, Dresden, Germany. [116]Merck Research Laboratories, Merck and Co., Inc., Boston, Massachusetts, USA. [117]Department of Clinical Sciences, Obstetrics and Gynecology, University of Oulu, Oulu, Finland. [118]National Institute for Health and Welfare, Department of Chronic Disease Prevention, Chronic Disease Epidemiology and Prevention Unit, Helsinki, Finland. [119]MRC Human Genetics Unit, Institute for Genetics and Molecular Medicine, Western General Hospital, Edinburgh, Scotland, UK. [120]Department of Psychiatry and Midwest Alcoholism Research Center, Washington University School of Medicine, St. Louis, Missouri, USA. [121]Klinik und Poliklinik für Innere Medizin II, Universität Regensburg, Regensburg, Germany. [122]Regensburg University Medical Center, Innere Medizin II, Regensburg, Germany. [123]Department of Child and Adolescent Psychiatry, University of Duisburg-Essen, Essen, Germany. [124]Interfaculty Institute for Genetics and Functional Genomics, Ernst-Moritz-Arndt-

University Greifswald, Greifswald, Germany. [125]PathWest Laboratory of Western Australia, Department of Molecular Genetics, J Block, QEII Medical Centre, Nedlands, Western Australia, Australia. [126]Busselton Population Medical Research Foundation Inc., Sir Charles Gairdner Hospital, Nedlands, Western Australia, Australia. [127]Division of Research, Kaiser Permanente Northern California, Oakland, California, USA. [128]Department of Epidemiology and Biostatistics, University of California, San Francisco, San Francisco, California, USA. [129]Department of Social Services and Health Care, Jakobstad, Finland. [130]Core Genotyping Facility, SAIC-Frederick, Inc., National Cancer Institute (NCI)-Frederick, Frederick, Maryland, USA. [131]Institute of Medical Biometry and Epidemiology, University of Marburg, Marburg, Germany. [132]Institut für Epidemiologie und Sozialmedizin, Universität Greifswald, Greifswald, Germany. [133]Research Centre for Prevention and Health, Glostrup University Hospital, Glostrup, Denmark. [134]Faculty of Health Science, University of Copenhagen, Copenhagen, Denmark. [135]National Institute for Health and Welfare, Department of Chronic Disease Prevention, Population Studies Unit, Turku, Finland. [136]Institute of Health Sciences, University of Oulu, Oulu, Finland. [137]Biocenter Oulu, University of Oulu, Oulu, Finland. [138]Hospital for Children and Adolescents, Helsinki University Central Hospital and University of Helsinki, Hospital District of Helsinki and Uusimaa (HUS), Helsinki, Finland. [139]Massachusetts General Hospital (MGH) Weight Center, Massachusetts General Hospital, Boston, Massachusetts, USA. [140]Cardiovascular Research Center and Cardiology Division, Massachusetts General Hospital, Boston, Massachusetts, USA. [141]Framingham Heart Study of the National, Heart, Lung, and Blood Institute and Boston University, Framingham, Massachusetts, USA. [142]Department of Medicine, Harvard Medical School, Boston, Massachusetts, USA. [143]National Institute for Health and Welfare, Diabetes Prevention Unit, Helsinki, Finland. [144]Department of Medicine, Stanford University School of Medicine, Stanford, California, USA. [145]Andrija Stampar School of Public Health, Medical School, University of Zagreb, Zagreb, Croatia. [146]Interdisciplinary Centre for Clinical Research, University of Leipzig, Leipzig, Germany. [147]Department of Medicine, University of Kuopio and Kuopio University Hospital, Kuopio, Finland. [148]Nord-Trøndelag Health Study (HUNT) Research Centre, Department of Public Health and General Practice, Norwegian University of Science and Technology, Levanger, Norway. [149]Finnish Institute of Occupational Health, Oulu, Finland. [150]Institut inter-regional pour la santé (IRSA), La Riche, France. [151]Laboratory of Epidemiology, Demography, Biometry, National Institute on Aging, National Institutes of Health, Bethesda, Maryland, USA. [152]Department of Clinical Chemistry, University of Tampere and Tampere University Hospital, Tampere, Finland. [153]Department of Medicine, Université de Montréal, Montreal, Quebec, Canada. [154]Human Genetics, Genome Institute of Singapore, Singapore, Singapore. [155]Transplantation Laboratory, Haartman Institute, University of Helsinki, Helsinki, Finland. [156]Department of Internal Medicine, Institute of Medicine, Sahlgrenska Academy, University of Gothenburg, Gothenburg, Sweden. [157]Department of Public Health and Primary Care, Institute of Public Health, University of Cambridge, Cambridge, UK. [158]On behalf of the MAGIC (Meta-Analyses of Glucose and Insulin-Related Traits Consortium) investigators. [159]Department of Endocrinology, Diabetology and Nutrition, Bichat-Claude Bernard University Hospital, Assistance Publique des Hôpitaux de Paris, Paris, France. [160]Cardiovascular Genetics Research Unit, Université Henri Poincaré-Nancy 1, Nancy, France. [161]Genetic Epidemiology Laboratory, Queensland Institute of Medical Research, Queensland, Australia. [162]Avon Longitudinal Study of Parents and Children (ALSPAC) Laboratory, Department of Social Medicine, University of Bristol, Bristol, UK. [163]Division of Health, Research Board, An Bord Taighde Sláinte, Dublin, Ireland. [164]Institute of Human Genetics, Klinikum rechts der Isar der Technischen Universität München, Munich, Germany. [165]Institute of Human Genetics, Helmholtz Zentrum München-German Research Center for Environmental Health, Neuherberg, Germany. [166]Department of Clinical Sciences, Lund University, Malmö, Sweden. [167]Molecular Epidemiology Laboratory, Queensland Institute of Medical Research, Queensland, Australia. [168]Croatian Centre for Global Health, School of Medicine, University of Split, Split, Croatia. [169]Neurogenetics Laboratory, Queensland Institute of Medical Research, Queensland, Australia. [170]National Heart, Lung, and Blood Institute, National Institutes of Health, Framingham, Massachusetts, USA. [171]University of Cambridge Metabolic Research Laboratories, Institute of Metabolic Science, Addenbrooke's Hospital, Cambridge, UK. [172]Department of Clinical Genetics, Erasmus MC, Rotterdam, The Netherlands. [173]Department of Pathology and Molecular Medicine, McMaster University, Hamilton, Ontario, Canada. [174]Amgen, Cambridge, Massachusetts, USA. [175]Finnish Twin Cohort Study, Department of Public Health, University of Helsinki, Helsinki, Finland. [176]Obesity Research Unit, Department of Psychiatry, Helsinki University Central Hospital, Helsinki, Finland. [177]Department of Medicine, Levanger Hospital, The Nord-Trøndelag Health

Trust, Levanger, Norway. [178]Gen-Info Ltd, Zagreb, Croatia. [179]National Institute for Health and Welfare, Oulu, Finland. [180]Department of Cardiovascular Sciences, University of Leicester, Glenfield Hospital, Leicester, UK. [181]Research Centre of Applied and Preventive Cardiovascular Medicine, University of Turku, Turku, Finland. [182]The Department of Clinical Physiology, Turku University Hospital, Turku, Finland. [183]Clinical Psychology and Psychotherapy, University of Marburg, Marburg, Germany. [184]Department of Clinical Sciences and Clinical Chemistry, University of Oulu, Oulu, Finland. [185]Ludwig-Maximilians-Universität, Institute of Medical Informatics, Biometry and Epidemiology, Chair of Epidemiology, Munich, Germany. [186]South Karelia Central Hospital, Lappeenranta, Finland. [187]Department of Clinical Sciences and Internal Medicine, University of Oulu, Oulu, Finland. [188]Institut für Community Medicine, Greifswald, Germany. [189]Christian-Albrechts-University, University Hospital Schleswig-Holstein, Institute for Clinical Molecular Biology and Department of Internal Medicine I, Kiel, Germany. [190]Universität zu Lübeck, Medizinische Klinik II, Lübeck, Germany. [191]Division of Cardiology, Cardiovascular Laboratory, Helsinki University Central Hospital, Helsinki, Finland. [192]Departments of Medicine and Epidemiology, University of Washington, Seattle, Washington, USA. [193]Department of Psychiatry, Instituut voor Extramuraal Geneeskundig Onderzoek (EMGO) Institute, VU University Medical Center, Amsterdam, The Netherlands. [194]Department of Haematology, University of Cambridge, Cambridge, UK. [195]National Health Service (NHS) Blood and Transplant, Cambridge Centre, Cambridge, UK. [196]Leicester NIHR Biomedical Research Unit in Cardiovascular Disease, Glenfield Hospital, Leicester, UK. [197]Department of Health Sciences, University of Leicester, University Road, Leicester, UK. [198]Department of Medicine, University of Leipzig, Leipzig, Germany. [199]Coordination Centre for Clinical Trials, University of Leipzig, Leipzig, Germany. [200]Department of Medicine, Helsinki University Central Hospital, Helsinki, Finland. [201]Research Program of Molecular Medicine, University of Helsinki, Helsinki, Finland. [202]Department of Human Genetics, Leiden University Medical Center, Leiden, The Netherlands. [203]Center of Medical Systems Biology, Leiden University Medical Center, Leiden, The Netherlands. [204]Department of Medicine, University of Turku and Turku University Hospital, Turku, Finland. [205]INSERM Cardiovascular Genetics team, Centre Investigation Clinique (CIC) 9501, Nancy, France. [206]Steno Diabetes Center, Gentofte, Denmark. [207]Center for Human Genomics, Wake Forest University, Winston-Salem, North Carolina, USA. [208]Department of Physiatrics, Lapland Central Hospital, Rovaniemi, Finland. [209]School of Pathology and Laboratory Medicine, University of Western Australia, Nedlands, Western Australia, Australia. [210]Department of Internal Medicine, University of Oulu, Oulu, Finland. [211]School of Medicine and Pharmacology, University of Western Australia, Perth, Western Australia, Australia. [212]Department of Clinical Physiology, University of Tampere and Tampere University Hospital, Tampere, Finland. [213]Stanford University School of Medicine, Stanford, California, USA. [214]Department of Psychiatry and Human Behavior, University of California, Irvine (UCI), Irvine, California, USA. [215]Leipziger Interdisziplinärer Forschungs-komplex zu molekularen Ursachen umwelt- und lebensstilassoziierter Erkrankungen (LIFE) Study Centre, University of Leipzig, Leipzig, Germany. [216]Service of Medical Genetics, Centre Hospitalier Universitaire Vaudois (CHUV) University Hospital, Lausanne, Switzerland. [217]Human Genetics Center and Institute of Molecular Medicine, University of Texas Health Science Center, Houston, Texas, USA. [218]Faculty of Health Science, University of Southern Denmark, Odense, Denmark. [219]New York University Medical Center, New York, New York, USA. [220]National Institute for Health and Welfare, Department of Mental Health and Substance Abuse Services, Unit for Child and Adolescent Mental Health, Helsinki, Finland. [221]NIHR Oxford Biomedical Research Centre, Churchill Hospital, Oxford, UK. [222]Department of Urology, Radboud University Nijmegen Medical Centre, Nijmegen, The Netherlands. [223]Institute of Biomedical Sciences, University of Copenhagen, Copenhagen, Denmark. [224]Faculty of Health Science, University of Aarhus, Aarhus, Denmark. [225]Department of Psychiatry, Leiden University Medical Centre, Leiden, The Netherlands. [226]Department of Psychiatry, University Medical Centre Groningen, Groningen, The Netherlands. [227]Department of Neurology, University of Lübeck, Lübeck, Germany. [228]Institute for Paediatric Nutrition Medicine, Vestische Hospital for Children and Adolescents, University of Witten-Herdecke, Datteln, Germany. [229]Department of Medicine III, Prevention and Care of Diabetes, University of Dresden, Dresden, Germany. [230]Geriatrics Research and Education Clinical Center, Baltimore Veterans Administration Medical Center, Baltimore, Maryland, USA. [231]Hjelt Institute, Department of Public Health, University of Helsinki, Helsinki, Finland. [232]South Ostrobothnia Central Hospital, Seinajoki, Finland. [233]Department of Internal Medicine, Centre Hospitalier Universitaire Vaudois (CHUV) University Hospital, Lausanne, Switzerland. [234]The Broad Institute of Harvard and Massachusetts Institute of Technology (MIT), Cambridge,

Massachusetts, USA. [235]Department of Psychiatry, Harvard Medical School, Boston, Massachusetts, USA. [236]Pacific Biosciences, Menlo Park, California, USA. [237]Sage Bionetworks, Seattle, Washington, USA. [238]Division of Biostatistics, Washington University School of Medicine, St. Louis, Missouri, USA. [239]Division of Intramural Research, National Heart, Lung, and Blood Institute, Framingham Heart Study, Framingham, Massachusetts, USA. [240]Department of Epidemiology and Population Health, Albert Einstein College of Medicine, New York, New York, USA. [241]Department of Genetics, University of North Carolina, Chapel Hill, North Carolina, USA. [242]Department of Medical Genetics, University of Helsinki, Helsinki, Finland. [243]Laboratory of Genetics, National Institute on Aging, Baltimore, Maryland, USA. [244]Division of Community Health Sciences, St. George's, University of London, London, UK. [245]Klinikum Grosshadern, Munich, Germany. [246]Faculty of Medicine, University of Iceland, Reykjavík, Iceland. [247]University of Cambridge Metabolic Research Labs, Institute of Metabolic Science Addenbrooke's Hospital, Cambridge, UK. [248]Carolina Center for Genome Sciences, School of Public Health, University of North Carolina Chapel Hill, Chapel Hill, North Carolina, USA. [249]Department of Genetics, Harvard Medical School, Boston, Massachusetts, USA.
